# Associations of health literacy with self-management behaviours and health outcomes in chronic kidney disease: a systematic review

**DOI:** 10.1007/s40620-022-01537-0

**Published:** 2023-01-16

**Authors:** Roseanne E. Billany, Ashnee Thopte, Sherna F. Adenwalla, Daniel S. March, James O. Burton, Matthew P. M. Graham-Brown

**Affiliations:** 1 Department of Cardiovascular Sciences, University of Leicester, Leicester, UK; 2 John Walls Renal Unit, University Hospitals of Leicester NHS Trust, Leicester, UK; 3 NIHR Leicester Biomedical Research Centre, University Hospitals of Leicester NHS Trust, Leicester, UK

**Keywords:** Health literacy, Chronic kidney disease, Self-management, End-stage kidney disease

## Abstract

**Introduction:**

Low health literacy is widely reported in people with chronic kidney disease (CKD) and has been associated with reduced disease self-management, poor health outcomes, increased mortality and poorer quality of life. However, these associations are still not well understood.

**Methods:**

Electronic-based systematic searches were performed to identify studies examining associations between health literacy and self-management behaviours and/or health outcomes in patients with CKD. A tabular and narrative synthesis of the data was performed. Meta-analysis was not appropriate due to the heterogeneity of study designs and methods.

**Results:**

Searches identified 48 studies that met the inclusion criteria. A total of 41 published articles, six conference abstracts, and one thesis were included. Of the 48 studies, 11 were cohort and 37 were cross-sectional. In total there were 25,671 patients; 16,952 from cohort studies. Median study sample size was 159 (IQR 92–275). Study quality was high (5), moderate (24) and poor (19). Thirteen measures of health literacy were used. Despite the limitations of the available evidence, there appear to be consistent relationships between higher health literacy and favourable self-management behaviours for patients with CKD. Definitive relationships between health literacy and patient outcomes are far less clear and remain incompletely understood.

**Discussion:**

Conclusive evidence describing a causal link between health literacy and patient outcomes remains limited, but for many outcomes, a consistent association is described. In addition to associations with mortality, hospitalisation and clinical events, there were consistent associations between health literacy and favourable self-management behaviours which could support the development of patient education aimed at improving health literacy.

**Graphical abstract:**

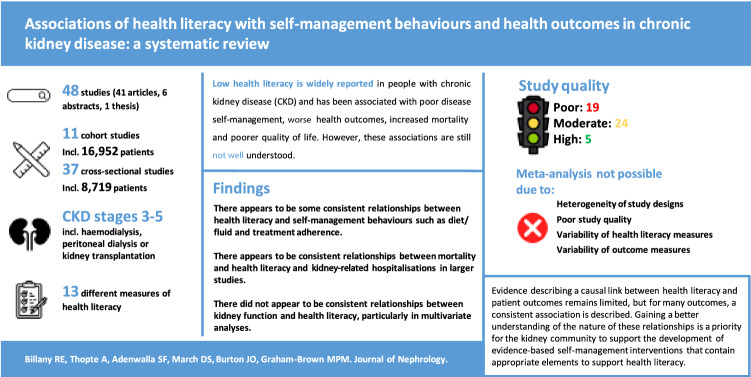

**Supplementary Information:**

The online version contains supplementary material available at 10.1007/s40620-022-01537-0.

## Introduction

The term health literacy refers to the cognitive and social skills, which establish the motivation and ability of an individual to gain access to, and to understand and use information in ways that promote good health, prevention of disease and improve quality of life (QoL) [1]. Early conceptualisations of health literacy focused on functional aspects, such as basic skills in reading and writing. In recent years, definitions and measures have evolved to include a more multidimensional approach encompassing behavioural and cognitive skills. Communicative health literacy represents the more advanced cognitive skills that allow information to be extracted and meanings derived [2]. Critical health literacy is the analysis of information and its application to achieve greater control over life events [3]. It is established that the health literacy of patients with chronic kidney disease (CKD) is often low and may limit the extent to which patients can effectively self-manage aspects of their health as well as directly or indirectly affect health outcomes [4]. The factors which contribute to health literacy are complex and interrelated, but link to education, income and socioeconomic background [5].

Self-management behaviours refer to the ability to learn and practice skills and behaviours which allow people to better manage their day-to-day health, and include behaviours related to medication adherence, physical activity, managing symptoms, information seeking and interaction with healthcare providers [1]. Health literacy and self-management behaviours are inherently linked, but account for and explain different aspects of patient experience. Gaining a greater understanding of the relationships between health literacy and self-management behaviours in patients with chronic illnesses will improve the way complex interventions are designed to account for the relative influence of these different factors on patient outcomes. This is especially true of patients with CKD, for whom the personal burdens of care are often high [6] and which leave many feeling overburdened and unable to be actively involved in decisions about their care or to appropriately engage with their treatment [4].

A previous systematic review evaluated the relationship between health literacy, mortality and patient outcomes in individuals with CKD [4]. Whilst there was no robust causal evidence linking health literacy and patient outcomes, associations between low health literacy and adverse health outcomes were evident.

This systematic review aimed to update and expand the previous review by examining the relationships between health literacy and self-management behaviours and health outcomes. Better defining these relationships will allow better planning of prospective studies and the design of complex interventions that seek to improve health literacy and self-management behaviours to improve patient experience and outcomes for people with CKD.

## Methods

### Protocol registration and eligibility criteria

**Fig. 1:**
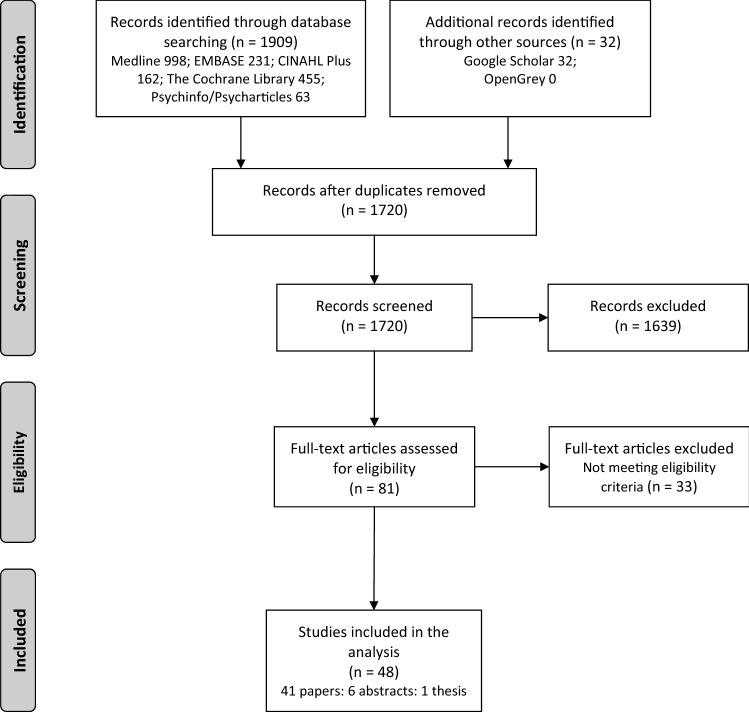
PRISMA flow diagram of identified studies for inclusion

The PRISMA flow diagram is shown in Fig. 1. This review was prospectively registered (PROSPERO 2020 reference: CRD42020201602).

Studies were considered eligible if they:

(i) Were retrospective, prospective, cross-sectional, longitudinal, case-control, cohort studies or randomised controlled trials (RCTs). Conference abstracts were included where sufficient data was present and data were not published elsewhere. Review articles, drug intervention studies, trial protocols, qualitative studies, and case reports were excluded, along with studies that were not in the English language.
(ii) Included adults, children, and adolescents with CKD stages three to five (estimated glomerular filtration rate (eGFR) less than 60 mL/min/1.73 m^2^) including those requiring renal replacement therapy (haemodialysis, peritoneal dialysis or kidney transplantation). Studies containing participants with CKD stages 1 and 2 exclusively were excluded.
(iii) Used an accepted tool for the assessment of health literacy (functional, communicative and/or critical health literacy).
(iv) Reported associations between health literacy and self-management behaviours and/or health outcomes as part of study outcomes.

### Search strategy

Electronic-based searches from the date of inception to December 2021 were performed to identify studies examining associations between health literacy and self-management behaviours and/or health outcomes in patients with CKD. The databases searched were: MEDLINE (Ovid), EMBASE (HDAS), CINAHL Plus (EBSCO), The Cochrane Library, PsycINFO (EBSCO), OpenGrey and Scopus. Trial registers searched included: ISRCTN Registry; ClinicalTrials.gov; WHO International Clinical Trials Registry Platform (ICTRP). Database searches were supplemented with internet searches (Google Scholar), contact with trial authors, experts and research groups, and identification of key citations from included trials and review articles. Search terms were adapted to database requirements and the full search strategy for the MEDLINE database is shown in supplementary material (appendix S1).

### Data extraction, synthesis and quality appraisal

Search results were managed using Endnote (Clarivate Analytics, Philadelphia, PA, USA) referencing software. Full texts were obtained and reviewed for articles meeting the inclusion criteria. Two reviewers independently reviewed two articles using the data extraction tool (an adapted version of the Cochrane Data Extraction Template) to ensure suitability. From therein, reviewers extracted data from each article including study design, sample-size, recruitment method, inclusion/exclusion criteria, primary aims, reported associations between health literacy and outcomes, and statistical testing. Crosschecking was performed by a second reviewer (R.E.B).

Titles and abstracts were screened by two reviewers (A.T and S.F.A) based on the criteria below. Agreement between the two reviewers was 86.28% (*k* = 0.354; 95% CI 0.292–0.416). Discrepancies were resolved with the inclusion of a third reviewer (R.E.B).

Study quality was independently assessed by three reviewers (D.S.M, S.F.A, and R.E.B) using a modified Newcastle Ottawa Scale, and final grading was decided following discussion. Quality scoring is presented within the supplementary material (appendix S2). Statistical significance was accepted as *p* < 0.05 unless individual studies stated otherwise.

Data are presented in tabular form including study characteristics, sample sizes, demographics of participants, health literacy measurement tools, outcome measures, associations tested between health literacy and self-management behaviours and health outcomes in univariate and multivariate analyses, covariates used, and any significant associations that were found. A narrative synthesis of findings, detailing the association between health literacy and self-management behaviours and/or health outcomes was also performed. Meta-analysis was not appropriate due to the heterogeneity of study designs and methods.

## Results

Searches identified 48 studies that met the inclusion criteria (Fig. 1). A total of 41 published articles, six conference abstracts, and one thesis were included. Of the 48 studies, 11 were cohort and 37 were cross-sectional. In total there were 25,671 patients; 16,952 from cohort studies. Median study sample size was 159 (IQR 92–275). Twenty-five studies included a total of 16,087 patients on haemodialysis, eight studies included 285 patients on peritoneal dialysis, eight studies included 791 kidney transplant recipients (KTRs), and nine studies included 3907 patients with non-dialysis CKD. Three studies described patients as having end-stage kidney disease (ESKD) or on dialysis but subgroup data were not available (2408 patients). Twelve studies included patients at multiple treatment stages but full subgroup data were unclear (2193 patients). No studies including paediatric patients were included. Detailed study characteristics of all studies included in the final synthesis are summarised within the supplementary material (appendix S3). Basic study characteristics are provided in Table 1 and an overview of the associations between health literacy and outcomes is provided in Table 2. Study quality was graded low for 19 studies, moderate for 24 studies, and high for 5 studies.

**Table 1:** Basic study characteristics grouped by health literacy measure

Study	Year	Design	CKD stage (or population)	*N*	Age (years) Median, IQR [mean, SD]	Male:female	Health literacy measure
Dahl et al. [44]	2020	Cross-sectional	KTR	159	58, 20–81	109:50	HLQ
Stømer et al. [18]	2020	Cross-sectional	CKD stages 3–5	187	[67, 13]	122:65	HLQ
Demian et al. [17]	2016	Cross- sectional	KTR	96	[53, 13]	54:42	HLQ
Gardiner [19]	2019	Cross-sectional	ESKD incl. KTR or waitlist	30	[48, 13]	16:14	HLQ
Murali et al. [40]	2020	Cross-sectional	HD and non-dialysis CKD	223	70, 63–74	136:87	HLQ
Dawson et al. [39]	2020	Cross-sectional	CKD 5 (PD, HD, conservative)	102	[73, 12]	69:33	HLQ
Dodson et al. [42]	2016	Cross-sectional	Dialysis (PD, HD)	100	68, 26–93	57:43	HLQ
Griva et al. [29]	2020	Prospective cohort	HD (+ diabetes)	221	[59, 10]	134:87	HLQ
Skoumalova et al. [20]	2019	Cross-sectional	HD	452	[64, 14]	329:123	HLQ – Slovak version
Zavacka et al. [49]	2020	Cross-sectional	HD	542	[64, 14]	329:213	HLQ – Slovak version
Lim et al. [22]	2019	Cross-sectional	HD	84	Not stated	Not stated	HLQ – European version
Cavanaugh et al. [30]	2010	Cohort	Incident HD	480	62, 51–72	269:211	REALM
Tohme et al. [26]	2017	Cohort	HD	286	64, 56–73	160:126	REALM
Wright Nunes et al. [23]	2015	Cross-sectional	CKD 1–5	155	[57, 15]	84:71	REALM
Jain et al. [33]	2015	Cross-sectional	PD	32	48 [13]	17:15	REALM
Patzer et al. [13]	2016	Cohort	KTR	99	53 [13.2]	66:33	REALM
Green et al. [41]	2011	Cross-sectional	HD	260	64, 56–73	163:97	REALM
Green et al. [28]	2013	Cohort	HD	260	62, 55–73	150:110	REALM
Wright et al. [11]	2011	Cross-sectional	CKD 1–5	401	58, 46–68	213:188	REALM
Cavanaugh et al. [48]	2010	Cross-sectional	HD	50	[51, 15]	24:26	REALM
Nelson et al. [16]	2015	Cross-sectional	CKD 3–5	208	[72]	116:92	REALM
Singla et al. [36]	2016	Cohort	CKD 3–4	74	[58, 13]	32:42	REALM
Schrauben et al. [10]	2020	Cross-sectional	CKD 1–5	401	[57, 16]	213:188	REALM
Balhara et al. [34]	2020	Cross-sectional with control	HD	49	Cases—[54, 13] Controls—[55, 11]	27:22	REALM-short form
Kazley et al. [43]	2014	Cross-sectional	Advanced CKD incl. KTR or dialysis	127	53 [17]	61:66	REALM—transplant Newest Vital Sign
Kazley et al. [46]	2015	Cross-sectional	Advanced CKD incl. KTR or dialysis	92	[54, 16]	47:45	REALM—transplant Newest Vital Sign
Gordon and Wolf [37]	2009	Cross-sectional	KTR	124	[47, 12]	70:54	REALM S-TOFHLA
Weng et al. [15]	2013	Cross-sectional	KTR	252	55, 45–63	151:101	S-TOFHLA
Ricardo et al. [24]	2014	Cross-sectional	CKD 2–4	2340	Limited HL [66, 9] Adequate HL [62, 11]	Limited HL 221:160 Adequate HL 1041:918	S-TOFHLA
Adeseun et al. [25]	2012	Cross-sectional	HD or PD	72	[51.6]	48:23	S-TOFHLA
Grubbs et al. [45]	2009	Cohort	HD	62	[52.4, 12.2]	41:21	S-TOFHLA
Foster et al. [59]	2011	Cross-sectional	HD or PD	311	[58, 15]	167:144	S-TOFHLA
Blandon et al. [38]	2011	Cross-sectional	CKD 2–4	225	49	110:115	S-TOFHLA
Dageford et al. [60]	2015	Cross-sectional	Scheduled for transplant evaluation	104	[52, 12]	63:41	Brief Health Literacy Screen
Warsame et al. [32]	2018	Cohort	Kidney transplant candidates	1578	[55, 13]	964:614	Brief Health Literacy Screen
Cavanaugh et al. [31]	2015	Cohort	HD	11,476	Not stated	Not stated	Brief Health Literacy Screen
Devraj et al. [9]	2015	Cross-sectional	CKD 1–4	150	Not stated Range 21–90	70:80	Newest Vital Sign
Devraj et al. [61]	2018	Cross-sectional	CKD 1–4	150	Not stated (range 21–90)	70:80	Newest Vital Sign
Mazarova et al. [47]	2017	Cross-sectional	HD	56	[63, 16]	35:21	Newest Vital Sign
Levine R et al. [35]	2018	Retrospective cohort	CKD 2–5 incl. KTR and dialysis	142	[21, 6]	89:53	Newest Vital Sign
Lai et al. [8]	2013	Cross-sectional	HD	63	[57, 10]	38:25	FCCHL
Indino et al. [21]	2019	Cross-sectional	HD	42	[54, 14]	25:17	FCCHL
Photharos et al. [12]	2018	Cross-sectional	CKD 2–3	275	Not stated > 70% were aged 51–65 years	165:110	Health Literacy Scale 14
Chen et al. [7]	2018	Cross-sectional	CKD 1–5	410	[70, 13]	259:151	S-MHLS
Yu et al. [62]	2021	Cross-sectional	CKD 1–5	208	[63.2, 12.8]	123:85	MMHLQ
Taylor et al. [27]	2019	Prospective cohort	Incident HD	2274	Limited HL 58, 47–66 Adequate HL 58, 47–67	Limited HL 231:128 Adequate HL 1243:672	Single Item Literacy Screener
Wong et al. [14]	2018	Cross-sectional	CKD 3–5	137	[55, 12]	66:71	Validated 3 item scale
Kita et al. [63]	2021	Cross-sectional	CKD 3–5	200	73 (61–80)	128:72	HLSEU-Q47

*CKD* chronic kidney disease, *ESKD* end stage kidney disease, *FCCHL* Functional, Communicative and Critical Health Literacy, *HD* haemodialysis, *HL* health literacy, *HLQ* Health Literacy Questionnaire, *HLSEU-Q47* Health Literacy Survey Questionnaire, *KTR* kidney transplant recipients, *MMHLQ* Mandarin Multidimensional Health Literacy Questionnaire, *PD* peritoneal dialysis, *REALM* Rapid Estimate of Adult Literacy in Medicine, *S-MHLS* Short-form Mandarin Health Literacy Scale, *STOFHL* Short Test of Functional Health Literacy in Adults

**Table 2:**
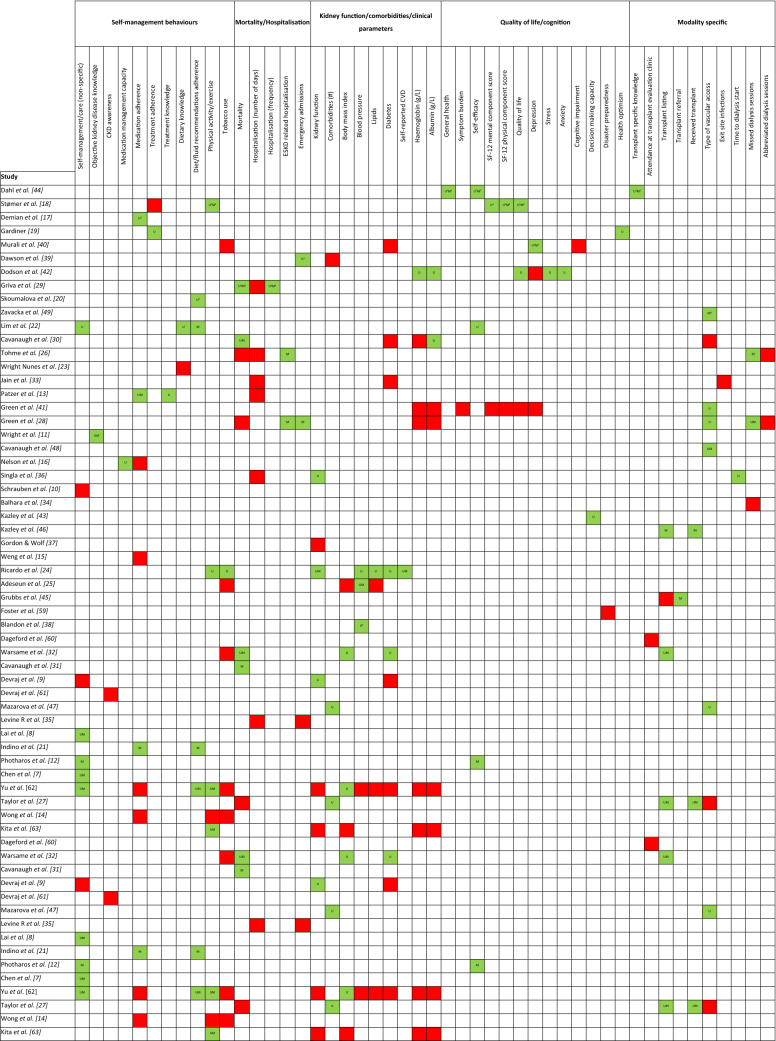
Basic overview of the associations between health literacy and self-management behaviours and health outcomes

^1^Significant in HLQ 9/9 domains, *P* < 0.2; ^2^significant in 7/9 HLQ domains; ^3^significant in 5/9 HLQ domains; ^4^significant in 1/9 HLQ domains; ^5^significant in 6/9 HLQ domains; ^6^significant in 2/9 HLQ domains; ^7^significant in 8/9 HLQ domains; ^8^significant in 4/9 HLQ domains; ^9^in a subgroup only (women with diabetes). Red signifies no significant association. Green signifies a significant association

### Health literacy measures

Table 3 describes the health literacy measures used within the reviewed studies, the classifications of adequate/limited health literacy, and the type of health literacy measured. Thirteen measures were used. The Health Literacy Questionnaire (HLQ), the Rapid Estimate of Adult Health Literacy in Medicine (REALM), and the Short Test of Functional Health Literacy in Adults (STOFHLA) were the most frequently used tools. The Brief Health Literacy Screen (BHLS), STOFHLA, and REALM were completed by the highest number of participants. Two tools (HLQ and the Functional Communicative Critical Health Literacy scale (FCCHL)) were not created to classify participants as having adequate or limited health literacy, but rather as separate scores for different sub-domains. The HLQ measures functional health literacy (domains 2, 8 and 9), communicative health literacy (domains 1, 3, 4, 6, 7 and 8) and critical health literacy (domains 3, 4 and 5).

**Table 3:** Health literacy measures used within the reviewed studies

Health literacy measure	Number of studies using the measure (%)	Total number of participants using the measure	Brief description of the measure	Type of health literacy measured	Health literacy categorisation
Health Literacy Questionnaire (HLQ)	11 (23)	2196	A multidimensional tool containing 44 items across nine independent scales that provides information about different dimensions of health literacy The nine domains of health literacy defined are: 1. Feeling understood and supported by healthcare providers 2. Having sufficient information to manage my health 3. Actively managing my health 4. Social support for health 5. Appraisal of health information 6. Ability to actively engage with healthcare providers 7. Navigating the healthcare system 8. Ability to find good health information 9. Understand health information well enough to know what to do	Functional Communicative Critical	The HLQ has no total summative score and no cut‐off for inadequate health literacy, however, higher scores indicate better health literacy in each domain
Rapid estimate of adult health literacy in medicine (REALM)	13 (27)	2570	125 health-related words (66 in more commonly used form) tested for pronunciation accuracy	Functional	0–44: inadequate 45–60: marginal 61–66: adequate (limited = inadequate + marginal)
REALM-SF	1 (2)	49	Short Form of REALM. Seven health-related words tested for pronunciation accuracy	Functional	0–3: inadequate 4–6: marginal 7: adequate
REALM-T	2 (4)	219	Transplant-specific version of REALM. 69 kidney transplant-related terms tested for pronunciation accuracy	Functional	0–44: inadequate 45–59: marginal 60–69: adequate
Newest Vital Sign (NVS)	6 (13)	440	Six-item assessment of reading comprehension from an ice-cream nutrition label	Functional	0–1: high likelihood marginal/inadequate 2–3: possible marginal/inadequate 4–6: adequate
Short test of functional health literacy in adults (STOFHLA)	7 (15)	3313	36 reading comprehension items—select from four choices to replace missing words in text (modified Cloze procedure)	Functional	0–16: inadequate 17–22: marginal 23–36: adequate
Brief Health Literacy Screen (BHLS)	3 (6)	13,158	A three-question subjective health literacy questionnaire answered on a five-point Likert scale	Functional	Limited (total score = 3–9) or adequate (total score = 10–15) health literacy (or limited health literacy ≤ 5 points and adequate health literacy > 5 points[32])
Functional Communicative Critical Health Literacy scale (FCCHL)	2 (4)	105	Five items for each sub-scale of functional and communicative health literacy, and four for critical health literacy, this 14-item self-report measure was rated on a range of 1–4 (never to often) for each item	Functional Communicative Critical	Mean scale scores obtained by reversing the item scores on each domain, summing them and dividing them by the total number of items, with scores ranging from 1 (limited health literacy) to 4 (high health literacy) for each domain. The FCCHL scales do not classify patients’ health literacy levels as adequate or inadequate
Health Literacy Scale-14 (HLS-14)	1 (2)	275	14 items with 5-point scales that indicate how much the respondent agrees or disagrees with the item (‘strongly disagree’ to ‘strongly agree’)	Functional Communicative Critical	The scores on the items are summed up to give the total health literacy score, as well as functional, communicative, and critical health literacy scores. Higher scores indicate a better health literacy
Short-form Mandarin Health Literacy Scale	1 (2)	410	11 items to assess functional health literacy in terms of the person’s ability to read, comprehend, and utilize basic health information when making individual health decisions	Functional	Total scores range from 0 to 11, with higher scores reflecting better health literacy
Single Item Literacy Screen (SILS)	1 (2)	2274	"How often do you need to have someone help you when you read instructions, pamphlets, or other written material from your doctor or pharmacy?" Possible responses are 1-Never, 2-Rarely, 3-Sometimes, 4-Often, and 5-Always	Functional	≤ 2 adequate health literacy 3–5 limited health literacy
Health Literacy Survey Questionnaire (HLSEU-Q47)	1 (2)	200	47 questions related to the domains of health care, disease prevention and health promotion, and the four competencies of health information access, understanding, evaluation, and utilization Response choices for each of the 47 questions are as follows: “Very easy” (four points), “Fairly easy” (three points), “Fairly difficult” (two points), “Very difficult” (one point), “Do not know/not applicable” (zero points)	Functional Communicative Critical	The scores of each item are summed to determine the total score Health literacy was categorised into the following four levels based on total score: “inadequate”, 0–25 points; “problematic” 25–33 points; “sufficient”, 33–42 points; and “excellent”, 42 points or more. “Inadequate” and “problematic” with 33 points or less were defined as “limited” [63]
Mandarin Multidimensional Health Literacy Questionnaire (MMHLQ)	1 (2)	208	20 self-reported items across 5 dimensions: accessing, understanding, appraising, and applying health information, and communication/interaction	Functional Communicative Critical	Total health literacy score = (sum of the average scores of the five dimensions—1) × 50/3. Health literacy is then graded as: inadequate (score range (SR): 0–25), limited/problematic (SR: 26–33), sufficient (SR: 34–42), and excellent (SR: 43–50)

### Self-management behaviours

Three cohort and 24 cross-sectional studies including a total of 5367 patients, explored elements of self-management. Study qualities were: 2 high, 15 moderate, and 10 low. Several studies reported on self-management behaviours as a whole, and others reported individual behaviours.

In 410 patients with CKD stages 1–5, health literacy was independently associated with self-management behaviours [7]. However, social support was the strongest determinant of behaviour. Total health literacy score was associated with diabetes self-management in 63 patients on haemodialysis [8]. In regression analysis, critical and communicative health literacy were associated with diabetes self-management and functional health literacy was not. Health literacy was not associated with self-management knowledge [9] or self-care behaviour scores [10] in 150 and 401 patients with non-dialysis CKD, respectively. After adjustment for demographics, CKD knowledge, CKD awareness, and education, lower health literacy was associated with lower kidney disease knowledge in 401 patients with non-dialysis CKD [11]. Health literacy had a moderate positive direct effect on self-management behaviours in 245 patients with CKD stages 2–3 [12].

#### Medical management

In a cohort study of 99 KTRs, limited health literacy was independently associated with higher odds of medication non-adherence [13]. Health literacy was not associated with medication adherence in 208 patients on haemodialysis, 252 KTRs, and 137 patients with non-dialysis CKD [14–16], but was associated with medication management capacity in cross-sectional analyses [16]. In three studies utilising the HLQ, health literacy was associated with medication adherence in 96 KTRs and 187 patients with non-dialysis CKD [17, 18] and with treatment adherence in 30 patients with ESKD [19]. In two of these studies, the domain ‘actively managing health’ was positively associated with medication adherence [17, 18].

#### Lifestyle

##### Diet

One study of 452 patients on haemodialysis reported that lower health literacy was associated with lower adherence to dietary and fluid intake recommendations [20]. Higher health literacy was associated with increased adherence to diet, fluid, and medication instruction in 42 patients on haemodialysis [21]. Health literacy, after adjustment for socioeconomic factors, was the only determinant of dietary adherence in 84 patients on haemodialysis and correlated with self-management skills [22]. In 155 patients with non-dialysis CKD, health literacy did not associate with dietary sodium knowledge [23]. Lower health literacy associated with lower intake of sugary drinks and lower fast food consumption in 137 patients with non-dialysis CKD after adjusting for demographics, education, and diabetes status [14].

##### Exercise

No association between health literacy and physical activity was shown in 137 patients with CKD stages 3–5 [14]. However, there was an association between higher health literacy and greater exercise habits in 200 patients with CKD stages 3–5 and an association between lower health literacy and lower total metabolic equivalent for physical activity (unadjusted analysis only in 2340 patients with CKD stages 2–4) [24].

##### Tobacco

Health literacy was not associated with tobacco use in 72 patients on dialysis [25], 137 patients with non-dialysis CKD [14], or 275 patients with CKD stages 2–3 [10].

### Mortality and hospitalisation

Seven cohort studies with a median sample size of 480 (IQR 260–2274) and follow-up times between 12 and 35 months examined the association between health literacy and mortality. One study was high quality and six studies were moderate quality. Six studies were of patients on haemodialysis and one study was of kidney transplant candidates. Three studies showed no association between health literacy and mortality in 2820 patients on haemodialysis [26–28]. The HLQ domain ‘actively managing my health’ was independently associated with mortality after adjusting for sociodemographic and clinical factors in 221 patients on haemodialysis [29]. One study reported an independent association between limited health literacy and increased mortality in patients on dialysis (HR 1.54; 95% CI 1.01–2.36), after adjustment for age, sex, race, and diabetes but not socio-economic status [30]. Similarly in 11,476 patients on dialysis, low health literacy was associated with increased mortality (HR = 1.65; 95% CI 1.28–2.12 Low vs. High) [31]. In 1578 kidney transplant candidates, there was an increased risk of waitlist mortality in those with limited health literacy [32]. This was robust to adjustment for income, comorbidity, and insurance type in a sensitivity analysis.

Eight studies (six cohort and two cross-sectional, 1216 patients) explored the association between health literacy and hospitalisation (median sample size 122, IQR 80–250). Study quality was: three low and five moderate. Lower hospitalisation rates were associated with the HLQ domain ‘actively managing my health’ in 221 patients on haemodialysis after adjustment for age, comorbidity, and education [29]. Lower health literacy was associated with ESKD-related hospitalisation but not total hospitalisation in an adjusted analysis of 286 patients on haemodialysis [26]. In a cross-sectional analysis of 102 patients with ESKD, higher scores on the HLQ domain ‘understanding health info well enough to know what to do’ were associated with lower emergency department admissions. One study showed no association between health literacy and hospitalisation in a cross-sectional analysis of 32 patients on peritoneal dialysis [33]. Another cross-sectional study showed no association between health literacy and missed dialysis sessions resulting in hospitalisation in 49 patients on haemodialysis [34]. Three cohort studies showed no association between health literacy and hospitalisation in patients with CKD stages 2–5 [35] (142 patients), 3–4 [36] (74 patients), and KTR [13] (99 patients).

### Kidney function, comorbidities and clinical parameters

Six studies (one cohort and five cross-sectional) totalling 3096 patients (median 175, IQR 112–741) explored the association between health literacy and kidney function. One study was low quality and five studies were moderate quality. In a cohort study of 74 patients with CKD stages 3–4, patients with inadequate or marginal health literacy had lower eGFR than those with adequate health literacy [36]. Limited health literacy was associated with lower eGFR in a cross-sectional analysis of 2340 patients with CKD stages 2–4 [24] and in 150 patients with CKD stages 1–4 [9]. This did not remain significant in the latter study after adjustment for age. In 124 KTRs, cross-sectional analysis did not describe any significant relationships between health literacy and eGFR [37]. However, health literacy was negatively correlated to serum creatinine after adjustment for time after transplant.

Cross-sectional analysis of 2340 patients with CKD stages 2–4 showed those with limited health literacy were more likely to report a history of cardiovascular disease after adjusting for age, gender, race, BMI, and education [24]. In unadjusted analysis, limited health literacy was associated with a reduced likelihood of having blood pressure below 130/80 mmHg and an increased prevalence of diabetes [24]. Adequate health literacy was associated with lower blood pressure and lower mean arterial pressure but not blood lipids after adjusting for both demographic and socioeconomic variables in 72 patients on dialysis [25]. In a cross-sectional analysis of 225 patients on dialysis, limited health literacy was associated with poor blood pressure control in women with diabetes but not in any other subgroup [38]. No health literacy domains were associated with the presence of comorbidities (< 3 or ≥ 3) in 102 patients with CKD stage 5 [39] or with the presence of diabetes in 223 patients with CKD [40].

### Quality of life and cognition

Ten cross-sectional studies explored QOL and/or outcomes relating to cognition with a total of 1643 patients (median 173, IQR 96–236). Four studies were moderate quality and six were low quality. In 187 patients with CKD stages 3–5, significantly better QOL was found in patients with higher health literacy [18]. However, in 260 patients on haemodialysis, QOL was not significantly different between those with adequate and those with limited health literacy [41]. This study did suggest depression may be worse in patients with limited health literacy, but this did not reach statistical significance. In 100 patients on dialysis, cross-sectional analysis showed that patients with higher health literacy reported fewer depressive symptoms [42]. Similarly, in 223 patients on dialysis and patients with non-dialysis CKD, depression was associated with worse health literacy in 8/9 domains on the HLQ [40]. Cognitive impairment was not associated with health literacy in this study. In unadjusted analyses, lower health literacy was associated with lower decision-making capacity in patients with advanced CKD [43]. Three cross-sectional studies showed an association between higher health literacy and high self-efficacy in KTR [44], patients on haemodialysis [22], and patients with CKD stages 2–3 [12].

### Modality-specific outcomes

Four studies (three cohort and one cross-sectional) evaluated the association between health literacy and aspects of the transplantation process. Study quality was: one high, two moderate, and one low. In a cohort study of 2274 patients receiving dialysis, limited health literacy was associated with a reduced likelihood of receiving a kidney transplant of any type but was not associated with pre-emptive transplant listing [27]. In a different study of 1578 kidney transplant candidates, limited health literacy was independently associated with a decreased likelihood of listing for transplantation [32]. In a cohort study of 62 patients on haemodialysis, lower health literacy was associated with a lower likelihood of being referred for transplant evaluation after controlling for race, gender, comorbidities, age, and support [45]. Higher health literacy was associated with transplant listing and receiving a transplant in a cross-sectional analysis of 92 patients with advanced CKD [46].

Seven studies (three cohort and four cross-sectional) explored the association of health literacy and vascular access. Study quality was: two high, three moderate, and two low. Patients with lower health literacy were more likely to choose a catheter over arteriovenous fistula (AVF) in a cross-sectional analysis of 56 patients on haemodialysis [47]. Similarly, catheter use was more common (versus AVF) in patients with limited health literacy after adjustment for age, gender, race, and dialysis vintage in a cross-sectional analysis [48]. In a cross-sectional analysis of 542 patients on haemodialysis, patients with a greater ability to engage with healthcare providers, those with a better ability to navigate the healthcare system, those more able to find good health information, and those who understood it well enough to know what to do were more likely to dialyse via an AVF [49]. Conversely, in a cohort study of 2274 patients on haemodialysis, limited health literacy was not associated with dialysis catheter use [27].

## Discussion

This systematic review synthesised data from 48 studies and 25,671 patients. There were more cross-sectional studies (*n* = 37) than cohort studies (*n* = 11), but cohort studies tended to be larger accounting for 16,952 of the patients included. Study quality was highly variable with only five studies classified as high quality. This variability, the heterogeneity of study designs and the limitations of statistical analyses mean it is not possible to define causal links between measures of health literacy and outcome measures assessed in this systematic review. The heterogeneity of the study designs and the wide variety of tools used to assess health literacy and outcomes meant it was also not possible to meta-analyse the available data. Fewer than one-quarter of studies assessed the relationships between health literacy and ‘hard’ outcomes such as mortality or hospitalisation, and there were limited numbers of studies that included KTRs or patients on peritoneal dialysis. No studies included paediatric patients. Whilst 27 studies explored the relationship between health literacy and self-management behaviours, only three of these were cohort studies. In a previous review of the relationships between health literacy and patient outcomes in patients with CKD [4], the majority of studies were only available as conference abstracts (13 articles; 16 conference abstracts). In the present review, there were 41 published articles (plus six abstracts; one thesis).

Within the limitations of the data available, there are consistent associations between health literacy, discrete self-management behaviours, and knowledge of CKD for patients with CKD, patients on haemodialysis and KTRs [7, 11, 13, 17–22]. For KTRs health literacy appears to associate most closely with health behaviours related to medication adherence [13]. This also seems to be the case for patients on haemodialysis [19] and those with CKD [17, 18]. These findings have several important implications for studies that seek to improve health-related behaviours such as medication adherence. Health literacy and self-management behaviours are inherently linked but describe different aspects of the patient experience. Even though the direction of causality between health literacy and health behaviours cannot be definitively defined from the available data, intuitively interventions that seek to improve self-management behaviours in patients with CKD will require carefully developed components that improve relevant aspects of health literacy. Defining the components of low health literacy for different populations of patients with kidney disease and between sub-groups of patients must be a priority for the kidney community to be able to support the development and implementation of appropriate self-management interventions. Our incomplete understanding of these issues and the relationships between health literacy and self-management behaviours may explain why there is only weak evidence that health literacy interventions in patients with CKD lead to meaningful improvements in knowledge, decision-making and self-care behaviours [50]. It is also clear that whilst appropriately designed, stand-alone interventions addressing aspects of health-related behaviours are needed, they do not have to be tested in isolation. Self-management interventions with appropriate components of health literacy could (and should) be developed to be tested alongside lifestyle interventions, pharmacological interventions and even interventions that involve medical devices. The inequities in access to participation in clinical trials for patients with kidney disease are well described at both an individual [51–53] and institutional level [54]. Embedding relevant health literacy and self-management interventions into clinical trials may go some way to improving recruitment and retention to trials for patients with low health literacy [55]. Appropriate designs would also allow testing of the effects of contemporaneous health literacy/self-management interventions alongside lifestyle, pharmacological or device-related interventions.

Whilst some studies suggest there is no clear link between health literacy and mortality for patients on haemodialysis [26–28], larger, prospective studies suggest that there is a link for these patients [30, 31], and for potential kidney transplant candidates [32]. Links between health literacy and mortality in patients with CKD and KTRs have not been well studied. Whilst smaller studies did not show clear links between health literacy measures and hospitalisations [13, 33–36], larger studies described a clear and consistent relationship for patients on haemodialysis [26, 29]. There were no data describing a consistent relationship between health literacy and kidney function, and it is not possible to draw any firm conclusions from data assessing possible relationships between health literacy and medical co-morbidities as the nature of the study designs and analyses do not account for confounding variables. There were no consistent associations described between health literacy and quality of life for patients on dialysis, KTRs or patients with CKD. There were clear and consistent associations between lower health literacy and a lower likelihood of being listed for a kidney transplant [27, 32, 45, 46], although whether this is independent of social or demographic factors is not clear. For patients on haemodialysis, whilst cross-sectional studies appear to describe a relationship between health literacy and likelihood of dialysing with an AVF [47, 48, 56], this finding was not corroborated by a larger cohort study [27], suggesting there is no clear link between health literacy and vascular access type.

Thirteen different health literacy questionnaires were used, with the REALM and the HLQ being the most common. The HLQ provides scores across nine independent health literacy domains, rather than a summative score, and therefore was not included in the previous systematic review [4]. However, its popularity has grown over recent years with the majority of the literature using this tool being published in 2019/20. The concept of health literacy has developed to become far more multidimensional, including social, critical, and interactive dimensions rather than just individual reading and numeracy skills [1, 57].

The strengths of this systematic review lie in the broad search strategy and the inclusion of paediatric studies. That there were no studies evaluating links between health literacy and self-management behaviours in paediatric patients with kidney disease is telling and identifies an immediate area for research. This review offers a significant update to the previous review [4], with data from over 7000 patients. Despite this, the findings of this review are broadly similar in that there remains significant heterogeneity in study design, in selection of outcome measures and in the way health literacy (and self-management behaviours) are assessed, thereby making a direct comparison between studies difficult and meta-analyses impossible. There are inherent limitations in the use of screening tools to assess health literacy, but they have been validated against comprehensive assessments [58]. Whilst there were fewer cohort studies than cross-sectional studies, there were more patients in cohort studies. Investigators should be encouraged to work collaboratively on large, multi-centre prospective studies. Consensus should be reached on the assessment of health literacy and of core outcomes to allow synthesis of studies and future meta-analyses and meta-regressions. Without consensus across these areas, the evidence base will remain piecemeal, inconclusive and limit our ability to inform the design of interventional studies of complex interventions.

## Conclusions

Conclusive evidence describing a causal link between health literacy and patient outcomes remains limited, but for many outcomes, a consistent association is described. In addition to associations with mortality, hospitalisation and clinical events, in this systematic review, we were also able to describe consistent associations between health literacy and self-management behaviours. Gaining a better understanding of the nature of these relationships is a priority for the kidney community to support the development of evidence-based interventions to support health literacy.

## Supplementary Information

Below is the link to the electronic supplementary material.

Supplementary file1 (DOCX 159 KB)

## Data Availability

Data sharing not applicable to this article as no datasets were generated or analysed during the current study.
