# Supplementary material for: Associations of health literacy with self-management behaviours and health outcomes in chronic kidney disease: a systematic review
Source: J Nephrol. 2023 Jan 16;36(5):1267–81. doi: 10.1007/s40620-022-01537-0 (PMC10333418; doi:10.1007/s40620-022-01537-0)
Supplement: Supplementary file 1 — Supplementary file1 (DOCX 159 KB) [file 40620_2022_1537_MOESM1_ESM.docx]

**Appendix S1**

Full search strategy for MEDLINE

1. exp Health Literacy/

2. (health adj3 litera*).mp

3. (literacy or literate).mp.

4. HL.mp.

5. Health Education/

6. Health Knowledge, Attitudes, Practice/

7. health knowledge.mp.

8. numeracy.mp.

9. Wide Range Achievement Test.mp.

10. Rapid Estimate of Adult Literacy in Medicine.mp.

11. Peabody Individual Achievement Test.mp.

12. Slosson oral reading test.mp.

13. National Adult Reading Test.mp.

14. (Woodcock-Johnson and test).mp.

15. literacy assessment for diabetes.mp.

16. adult basic education test.mp.

17. Newest Vital Sign.mp.

18. Short Assessment of Health Literacy.mp.

19. Health literacy Screening Question Methodologies.mp.

20. Single-Item Literacy Screener.mp.

21. Health Literacy Skills Instrument.mp.

22. Medical Term Recognition Test.mp.

23. Short Literacy Survey.mp.

24. Brief Health literacy Screen.mp.

25. Short test of functional health literacy in adults.mp.

26. SORT.mp.

27. REALM.mp.

28. MART.mp.

29. TOFHLA.mp.

30. STOFHLA.mp.

31. WRAT.mp.

32. PIAT.mp.

33. NART.mp.

34. AMNART.mp.

35. NVS.mp.

36. SAHLSA.mp.

37. HLSQM.mp.

38. SILS.mp.

39. HLSI.mp.

40. HLSI-SF.mp.

41. METER.mp.

42. SAHL-S&E.mp.

43. SLS.mp.

44. BHLS.mp.

45. STOFHLA.mp.

46. exp Kidney Diseases/

47. (kidney disease* or renal disease* or kidney failure or renal failure).mp.

48. exp Renal Insufficiency/

49. renal insufficiency.mp.

50. Renal Insufficiency, Chronic/

51. exp Renal Replacement Therapy/

52. renal replacement therapy.mp.

53. Dialysis/

54. (hemodialysis or haemodialysis).mp.

55. (hemofiltration or haemofiltration).mp.

56. (hemodiafiltration or haemodiafiltration).mp.

57. peritoneal dialysis/

58. (peritoneal and dialysis).mp.

59. (ESRF or ESKF or ESRD or ESKD).mp.

60. (CKF or CKD or CRF or CRD).mp.

61. (CAPD or CCPD or APD).mp.

62. (predialysis or pre-dialysis).mp.

63. exp Kidney Transplantation/

64. (renal transplant* or kidney transplant*).mp.

65. 4 or 5 or 6 or 7 or 8

66. 3 and 65

67. 9 or 10 or 11 or 12 or 13 or 14 or 15 or 16 or 17 or 18 or 19 or 20 or 21 or 22 or 23 or 24 or 25

68. 26 or 27 or 28 or 29 or 30 or 31 or 32 or 33 or 34 or 35 or 36 or 37 or 38 or 39 or 40 or 41 or 42 or 43 or 44 or 45

69. 46 or 47 or 48 or 49 or 50 or 51 or 52 or 53 or 54 or 55 or 56 or 57 or 58 or 59 or 60 or 61 or 62 or 63 or 64

70. 1 or 2 or 66 or 67 or 68

71. 69 and 70

**Appendix S2**

Quality assessment tools

Cohort Studies

Cross-sectional studies

**Scores**

Cohort

High 8-9
Mod 5-7
Low 0-4

| Study | Score (9) | Selection (4) | Comparability (2) | Outcome (3) |
| --- | --- | --- | --- | --- |
| Cavanaugh, Wingard, et al. (2010) | 6 | 3 | 2 | 1 |
| Griva et al. (2020) | 6 | 2 | 2 | 2 |
| Patzer et al. (2016) | 6 | 2 | 2 | 2 |
| Singla et al. (2016) | 3 | 1 | 0 | 2 |
| Taylor et al. (2019) | 8 | 3 | 2 | 3 |
| Warsame et al. (2019) | 7 | 3 | 2 | 2 |
| Grubbs et al. (2009) | 7 | 3 | 2 | 2 |
| Cavanaugh KL et al. (2015) | 6 | 2 | 2 | 2 |
| Green et al. (2013) | 7 | 3 | 2 | 2 |
| Levine et al. (2018) | 7 | 4 | 0 | 3 |
| Tohme et al. (2017) | 7 | 3 | 2 | 2 |

Cross-sectional

High 7-8
Mod 5-6
Low 0-4

| Study | Score (8) | Selection (4) | Comparability (2) | Outcome (2) |
| --- | --- | --- | --- | --- |
| Adeseun, Bonney, and Rosas (2012) | 4 | 0 | 2 | 2 |
| Stomer et al. (2020) | 6 | 2 | 2 | 2 |
| Balhara et al. (2020) | 2 | 1 | 0 | 1 |
| Cavanaugh, Rothman, et al. (2010) | 3 | 0 | 2 | 1 |
| Chen et al. (2018) | 7 | 3 | 2 | 2 |
| Dageforde et al. (2015) | 1 | 0 | 1 | 0 |
| Demian, Shapiro, and Thornton (2016) | 7 | 3 | 2 | 2 |
| Devraj et al. (2015) | 6 | 2 | 2 | 2 |
| Devraj et al. (2018) | 6 | 2 | 2 | 2 |
| Gordon and Wolf (2009) | 5 | 1 | 2 | 2 |
| Green et al. (2011) | 5 | 2 | 1 | 2 |
| Indino, Sharp, and Esterman (2019) | 4 | 2 | 1 | 1 |
| Kazley (2015) | 4 | 1 | 1 | 2 |
| Kazley et al. (2014) | 2 | 1 | 0 | 1 |
| Lai et al. (2013) | 3 | 2 | 0 | 1 |
| Nelson (2015) | 1 | 0 | 0 | 1 |
| Photharos, Wacharasin, and Duongpaeng (2018) | 5 | 2 | 1 | 2 |
| Ricardo et al. (2014) | 6 | 2 | 2 | 2 |
| Schrauben et al. (2020) | 5 | 1 | 2 | 2 |
| Wong et al. (2018) | 5 | 2 | 2 | 1 |
| Wright et al. (2011) | 6 | 3 | 1 | 2 |
| Zavacka et al. (2020) | 7 | 3 | 2 | 2 |
| Murali et al. (2020) | 5 | 1 | 2 | 2 |
| Dawson et al. (2020) | 4 | 2 | 0 | 2 |
| Dodson (2016) | 3 | 1 | 0 | 2 |
| Foster et al. (2011) | 4 | 2 | 0 | 2 |
| Gardiner (2019) | 3 | 1 | 0 | 2 |
| Jain et al. (2015) | 2 | 0 | 0 | 2 |
| Mazarova et al. (2017) | 3 | 1 | 0 | 2 |
| Weng et al. (2013) | 5 | 1 | 2 | 2 |
| Saunders et al. (2017) | 7 | 3 | 2 | 2 |
| Skoumalova et al. (2019) | 6 | 2 | 2 | 2 |
| Wright Nunes et al. (2015) | 6 | 2 | 2 | 2 |
| Dahl et al. (2020) | 4 | 0 | 2 | 2 |
| Kita et al. (2021) | 5 | 1 | 2 | 2 |
| Yu et al. (2021) | 5 | 1 | 2 | 2 |
| Lim et al. (2019) | 4 | 1 | 1 | 2 |

**Appendix S3**

Table X. Study characteristics grouped by health literacy measure

| Author/  Year /  Study design/  *N* | Patient Characteristics  CKD stage (or population)/  Exclusion Criteria | Age (years)  Median, IQR  [mean, SD] | Male: Female | Health literacy measure | Main aim | Outcome variables  (relating to HL) | Key findings |
| --- | --- | --- | --- | --- | --- | --- | --- |
| Dahl *et al.[1]*  2020  Cross-sectional  159 | KTR  ≤18 years, contagious diseases, unable to participate in patient education post-transplantation | 58, 20-81 | 109:50 | Health Literacy Questionnaire (HLQ) | Identify core variables associated with independent domains of HL 8 weeks  following a kidney transplant | Transplant knowledge (Knowledge Questionnaire for Renal Recipients)  Self-efficacy (General Perceived Self-Efficacy (GSE))  General health score | General perceived self-efficacy, transplant-specific knowledge, and general health were the driving variables in several of the HL domains |
| Stømer *et al.[2]*  2020  Cross-sectional  187 | CKD stages 3-5  Patients with non-cutaneous cancer, unstable CVD, a significant vascular incident  or major surgery <3 months | [67, 13] | 122:65 | Health Literacy Questionnaire (HLQ) | Explore the relationship between multidimensional HL, QoL, and adherence to long‐term therapy | SF-12 physical component score  SF-12 mental component score  VAS-QoL global quality of life  Adherence to long‐term therapy  Medical adherence report scale-5 (MARS-5)  Visual analogue scale-adherence (VAS-adherence) | Patients were divided into three groups: low, mid, and high HL  Significantly better QoL (SF‐12 and VAS‐QoL) was found in the high‐level group compared with the low‐level group  The VAS‐adherence scores were significantly higher in the high v mid group. The low group was not significantly different from the other groups The MARS‐5 scores did not differ among the groups  Collection of prescribed medication was not significantly different among the groups  The HLQ9, “ability to understand health information well enough to know what to do,” was positively associated with the PCS‐12 and VAS‐QoL  HLQ3, “actively managing health” and scale number 6, “ability to actively engage with healthcare providers” were positively associated with the VAS‐adherence |
| Demian *et al.[3]*    2016  Cross sectional  96 | KTR  Not capable of giving informed consent, impairments in vision, hearing, or other sensory or motor functions, Non-English language fluency, <sixth-grade education, psychosis, major neurological disease, other current major organ failure, ≤19 | [53, 13] | 54:42 | Health Literacy Questionnaire (HLQ) | To examine the role of HL in medication adherence | Self-reported medication adherence (Transplant Effects Questionnaire - TxEQ) | After accounting for age, gender, employment status and depressive symptoms, decreased total HL was associated with decreased medication adherence (ΔR2 = 0.08, P=.004)  The overall model explained 19% of the variance in adherence (95% CI 0.03–0.31, P=.002)  Social support, sufficient information, reading and understanding health information, navigating healthcare system, active engagement, healthcare provider support were correlated with self-reported medication adherence |
| Gardiner[4]  2019  Cross-sectional  30  [thesis] | ESKD (including RTR or waitlist)  Unable to read English, <18 | [48, 13] | 16:14 | Health Literacy Questionnaire (HLQ) | The effects of HL on treatment adherence and health optimism | P1Ank Sum Scale for treatment adherence  Life Orientation Scale for health optimism | There was a positive, strong correlation between HL and treatment adherence (M=3.17, SD=1.23), r= .584, P=. 001  There was a positive correlation between the HL and health optimism (M=14.97, SD=4.08), r= .445, P=.016 |
| Murali *et al.[5]*  2020  Cross-sectional  223 | Haemodialysis and non-dialysis CKD  Non-English speaking, KTR, working up for a living donor transplant, dementia or an intellectual impairment, life expectancy <12 months, renal palliative care pathway, <18 | 70, 63-74 | 137:96 | Health Literacy Questionnaire (HLQ) | To directly compare the HL profile of patients with ESKD undergoing dialysis with patients with non-dialysis CKD | HL domains in relation to:  Depression (Beck Depression Inventory 2 – BDI-2)  Diabetes presence  Cognitive impairment (Mini-Mental State Examination) | Depression was associated with worst HL in 8/9 domains  Cognitive impairment was not associated with HL in any domain and neither was diabetes presence |
| Dawson *et al.[6]*  2020  Cross-sectional  102 | CKD 5 (peritoneal dialysis, haemodialysis, conservative care)  Acutely illness, withdrawal from dialysis or being palliated, diagnosed with dementia, written language unavailable in the HLQ | [73, 12] | 69:33 | Health Literacy Questionnaire (HLQ) | To examine the HL of people with ESKD | Co-morbidities  ED admissions (last 12 months) | No HL domains were associated with presence of comorbidities (<>3)  Only ‘understanding health information well enough to know what to do’ was associated with ED admissions (P=.013) |
| Dodson *et al.[7]*  2016  Cross-sectional  100 | Dialysis (haemodialysis (n = 76), home haemodialysis (n = 8), peritoneal dialysis (n = 16))  Non-English speaking, hospital admission, cognitive impairment | 68, 26-93 | 57:43 | Health Literacy Questionnaire (HLQ) | Examine the relationships between HL, QoL and psychological quality | Kidney Disease Quality of Life-36 (KDQoL-36)  Depression Anxiety Stress Scales-21 (DASS-21) | Higher HL cluster demonstrated higher serum albumin than those in the lower HL cluster (mean difference = 2.1 g/L, p=.04) and lower levels of reported depressive symptoms (mean difference = 4.72 points on the DASS-21 Depression scale, p=.02) and anxiety symptoms (mean difference = 2.85 points on the DASS-21 Anxiety scale, p=.05  Differences were also observed on four of the five components of the KDQoL-36, with the higher HL cluster reporting better management of their kidney disease’s effect on daily life (mean difference = 13.07 points on  the KQQOL-36 effect on daily life scale, p<.01), burden (mean difference = 11.75 points on the KQQOL-36 burden of kidney disease scale, p<.01), physical aspects (mean difference = 5.82 points on the KQQOL-36 physical components scale, p=.01), and mental burden (mean difference = 5.89 points on the KQQOL-36 mental component scale, p=.01) |
| Griva *et al.[8]*  2020  Prospective cohort  221 | Haemodialysis (+diabetes)  Haemodialysis <3 months inability to communicate in either English, Mandarin, or Malay, speech difficulties or severe cognitive impairment, including dementia | [59, 10] | 134:87 | Health literacy questionnaire (HLQ) | To evaluate the broader range of HL skills, to identify the factors associated with different HL skills, and to prospectively examine their prognostic associations with hospitalization and mortality against other known risk factors, that is, age, comorbidity, and depression | All-cause hospitalisation (i.e., number of events and hospitalisation days)  All‐cause mortality [study enrolment to end of follow‐up; 21.8±3.2 months] | Among the HLQ domains, (univariate) only Actively Managing my Health was significantly associated with (lower) hospitalization rates  In the adjusted multivariable model (adjusting for age, comorbidity, and education) Actively Managing my Health remained independently associated with hospitalization rates  In the adjusted multivariable model, only the HLQ domain Actively managing my health remained independently associated with mortality risk after controlling for socio‐demographic and clinical variables |
| Skoumalova *et al.[9]*  2019  Cross-sectional  452 | Haemodialysis  Haemodialysis <90 days, inability to complete questionnaires, acute severe intercurrent illness, presence of a psychiatric diagnosis, <18 | [64, 14] | 329:123 | Health Literacy Questionnaire (HLQ) - Slovak version | To assess the associations of multidimensional HL with non-adherence to dietary and fluid intake recommendations | Associations between HL and non-adherence to dietary and fluid recommendations | Higher non-adherence for dietary and fluid recommendations among patients with lower HL  The patients with less sufficient information for managing health (HLQ2) were more likely to have high serum phosphate levels (OR: 0.77; 95% CI: 0.63–0.94)  Those with a lower ability to actively manage their health (HLQ3) were more likely to report non-adherence to diet recommendations (OR: 0.74; 95% CI: 0.62–0.89)  Those less able to actively engage with healthcare providers (HLQ6) were more likely to be overhydrated (OR: 0.78; 95% CI: 0.65–0.94) |
| Zavacka *et al.[10]*  2020  Cross-sectional  542 | Haemodialysis  Haemodialysis <90 days, inability to complete questionnaire (dementia, mental retardation, psychiatric diagnosis, inability to speak and read in  Slovak language and those with acute severe intercurrent illness), <18 | [64, 14] | 329:213 | Health Literacy Questionnaire (HLQ) - Slovak Version | To explore the association of HL and the type of vascular access | Vascular access (VA) | Four domains of health literacy were significantly associated with type of vascular access (more likely to have arteriovenous fistula):  Ability to actively engage with healthcare providers (OR 1.34 (OR 1.00-1.78), P<.05). Navigating the healthcare system (OR 1.41 (1.08-1.85), P<.05). Ability to find good health information (OR 1.52 (1.15-2.03), P<.01). Understand health information well enough to know what to do (OR 1.52 (1.12-2.06), P<.01) |
| Lim *et al.[11]*  2019  Cross-sectional  84  [abstract only] | Haemodialysis | Not stated | Not stated | Health Literacy Questionnaire - European Version (HLS-EU-Q) | To investigate HL in the context of dietary knowledge, health belief, self-management skill and dietary adherence | Dietary adherence Dietary knowledge  Health Belief Model | Multivariate analysis indicated HL was the only predictor of dietary adherence (beta=0.899, P=.004) after adjusting for sociodemographic factors  HL was significantly correlated (all P<.001) with dietary knowledge (r=0.704), perceived benefits (r=0.408), perceived barriers (r=-0.435), perceived self-efficacy (r=0.531) and self-management skills (r=0.691) but not with perceived seriousness and perceived susceptibility (both P>.05) |
| Cavanaugh *et al. [12]*  2010  Cohort  480 | Incident haemodialysis  Seasonal or transient patients, a known diagnosis of severe cognitive impairment, or dementia, non-English speaking, <18 | 62, 51-72 | 269:211 | Rapid Estimate of Adult Literacy in Medicine (REALM) | To describe the prevalence of limited HL and its association with the risk of all-cause mortality | Mortality [14-35 month follow-up]  Laboratory measures [12 months] | Compared with adequate literacy, limited HL associated with a higher risk for death (HR 1.54; 95% CI 1.01 to 2.36) even after adjustment for age, sex, race, and diabetes.  There was a difference in baseline serum albumin by HL, but no significant difference by HL level in the laboratory measures of Kt/V, haemoglobin, transferrin saturation, phosphorus, calcium, calcium-phosphorus product, or intact-parathyroid hormone at 3, 6, or 12 months after dialysis initiation |
| Tohme *et al.[13]*  2017  Cohort  286 | Haemodialysis  >18, awaiting living kidney donor transplant, considering transfer to peritoneal dialysis, cognitive impairment and non-English speaking, <18 | 64, 56-73 | 160:126 | Rapid Estimate of Adult Literacy in Medicine (REALM) | To examine the associations of patients’ demographic, socioeconomic, and clinical characteristics with non-adherence to dialysis-related therapy  Secondary aim to evaluate the associations of non-adherent behaviour with hospitalizations and mortality | Non-adherence to dialysis related therapy  e.g. missed/ abbreviated dialysis sessions [24 months]  Hospitalisation  Mortality | Lower baseline HL was independently associated with missed dialysis sessions (IRR 3.01, P<.01)  Limited HL IRR 1.20 (0.83-1.74) p=.33 for abbreviated dialysis session  Limited HL was not associated with total hospitalisations (IRR 1.11 P=.05) or mortality (HR 0.95 P=.91) but was associated with ESKD-related hospitalisations (IRR 1.55 P=.03)  Confounders: age, sex, dialysis vintage, type of vascular access, dialysis schedule, smoking, pain score, HL, race, comorbid illness burden, marital status, HRQoL |
| Wright Nunes *et al.[14]*  2015  Cross-sectional  155 | CKD 1-5  eGFR >60 ml.min.1.73m^2^, dialysis, KTR, Non-English speaking | [57, 15] | 84:71 | Rapid Estimate of Adult literacy in Medicine (REALM) | To identify factors relating to dietary sodium knowledge | Short Sodium Knowledge Survey (SSKS) | Inadequate/adequate HL had no association with dietary sodium knowledge (OR 2.27 [0.86,5.98]; P=.10) |
| Jain *et al.[15]*  2015  Cross-sectional  32 | Peritoneal dialysis  <18 | 48 [13] | 17:15 | Rapid Estimate of Adult Literacy in Medicine (REALM) | Characterise the prevalence of limited HL and assess its associations with key infectious complications and hospitalisations | Exit-site infections (ESI)  Peritonitis  Hospitalisations | Compared with patients with adequate HL, patients with limited HL had similar annual incident rates of ESI (IRR =  0.0 vs 0.17, P=.11), peritonitis (IRR = 0.21 vs 0.15, P=.55), and hospitalisations (IRR = 1.14 vs 0.82, P=.23) |
| Patzer *et al.[16]*  2016  Cohort  99 | KTR  Non-English speaking, severe cognitive, hearing, or vision impairment, transplant <30 days, <18 | 53 [13.2] | 66:33 | Rapid Assessment of Adult Literacy in Medicine (REALM) | Evaluate both patient and regimen‐specific risk factors for non‐adherence, treatment knowledge, and demonstrated regimen use  Examine associations between these behaviours and post‐transplant outcomes, including hospitalisation, acute rejection, and infection | Treatment knowledge  Demonstrated regimen use  Non-adherence by tacrolimus level or self-report (Patient Medication Adherence Questionnaire)  Number of hospitalisations at 12 months | Limited HL (β=−10.44; 95% CI: −18.72, −2.15; P<.05) was associated with lower treatment knowledge scores, but not in the multivariate analysis  Those with limited vs adequate HL (OR=2.93; 95% CI: 1.13, 7.56; P<.05) were also more likely non‐adherent    Limited HL (OR=2.80, CI: 1.02, 7.53) was independently associated with higher odds of medication non-adherence (P<.05)  Limited HL was not a significant predictor of number of hospitalisations at 12 months |
| Green *et al.[17]*    2011  Cross-sectional  260 | Haemodialysis  <17, non-English-speaking, undergoing live-donor work-up, planned change of modality, cognitive impairment | 64, 56-73 | 163:97 | Rapid Estimate of Adult Literacy in Medicine (REALM) | To characterise associations between HL and HRQoL, symptom burden and depression | HRQoL  Symptom burden  Depression | Depression worse in patients with low HL but not significantly (P=.17) and QoL and symptom burden were similar in patients with adequate and limited HL (P=.93 and P=.86, respectively)  No associations between age, gender, haemoglobin (Hb), phosphate, parathyroid hormone (PTH), dialysis adequacy and HL |
| Green *et al.[18]*  2013  Cohort  260 | Haemodialysis  Non-English speakers, cognitive dysfunction, those considering transfer  to peritoneal dialysis therapy and/or awaiting living donor kidney transplantation, <18 | 62, 55-73 | 150:110 | Rapid Estimate of Adult Literacy in Medicine (REALM) | To assess  the associations of limited HL with dialysis  adherence and  health resource utilisation  Associations of HL with kidney transplantation | Missed dialysis treatments  Abbreviated dialysis treatments  Emergency department visits  ESKD-related hospitalisations  Mortality [24 months] | In unadjusted and adjusted analyses, limited HL was associated with an increased incidence of missed dialysis treatments but not abbreviated treatments  In unadjusted analyses, there was a borderline association of limited HL with increased emergency department visits and ESRD-related hospitalizations. In adjusted models, limited HL was associated independently with increased emergency department visits and ESRD-related hospitalisations  No association between limited HL and receipt of a kidney transplant or mortality  Confounding variables: age, sex, race, employment, income, comorbid illness burden, dialysis vintage in years, type of vascular access, and randomization group |
| Wright *et al.[19]*  2011  Cross-sectional  401 | CKD 1-5  Non-English speaking, kidney transplant recipient or dialysis, cognitive or vision impairment, <18 | 58, 46-68 | 213:188 | Rapid Estimate of  Adult Literacy in Medicine (REALM) | Awareness and knowledge of CKD  Development and Results of a Kidney Disease Knowledge  Survey Given to Patients With CKD | Kidney knowledge | Low HL associated with lower kidney disease knowledge (P=.001)  In multivariable analysis adjusted for age, CKD stage,  education, HL level, participation in a  kidney education class, knowing someone with CKD,  and awareness of CKD diagnosis, higher HL remained associated with kidney disease knowledge (β =.05; 95% CI, 0.01-0.09; P=.04) |
| Cavanaugh *et al.[20]*  2010  Cross-sectional  50  [abstract only] | Haemodialysis | [51, 15] | 24:26 | Rapid Estimate of Adult Literacy in Medicine (REALM) | To examine the association between HL and type of dialysis access used | Catheter use or arteriovenous fistula (AVF) or graft | Catheter (vs. AVF or graft) use was more common in those with limited compared to adequate HL (53% vs. 23%; P=.04)    Patients with limited HL were almost 5 times more likely to use a catheter for dialysis access compared to those with adequate HL even after adjustment for age, gender, race, and years of dialysis (OR (95% CI): 4.8 (1.0 - 24.0); P=.05) |
| Nelson *et al.[21]*  2015  Cross- sectional  208  [abstract only] | CKD 3-5  Haemodialysis | [72] | 116:92 | Rapid Estimate of Adult Literacy in Medicine (REALM) | To explore the association of HL with medication management capacity and medication adherence | Medication management capacity  Medication adherence | Patients with limited HL had lower medicines management capacity (97 vs. 100, P<.001)  There was no association between HL and medication adherence (P=.73) |
| Singla *et al.[22]*  2016  Cohort  74  [abstract only] | CKD 3-4 | [58, 13] | 32:42 | Rapid Estimate of Adult Literacy in Medicine (REALM) | To identify the prevalence of and factors associated with low HL to identify high-risk individuals | eGFR  Dialysis initiation [2 years follow-up]  Hospitalisation [2 years follow-up] | Patients with inadequate (mean eGFR 28) and marginal (mean eGFR 33) HL had lower eGFRs compared to those with adequate HL (mean eGFR 39) (P=.03)  There was no association between HL and hospitalisations  There were only 2 patients who initiated dialysis over the 2 year period. Both had inadequate HL |
| Schrauben *et al.[23]*  2020  Cross-sectional  401 | CKD 1-5  Cognitive/visual impairments, KTK, dialysis treatment, <1 visit to a nephrologist, non-English speaking | [57, 16] | 213:188 | Rapid Estimate of Adult Literacy in Medicine (REALM) | To describe the association of 2 types of kidney disease knowledge (perceived and objective), HL, and self-care behaviours | Self-care behaviours | Median CKD self-care summary scores did not differ  significantly between those with inadequate and adequate HL (24.7 [21.3–26.8] vs. 25.7 [22.7–27.8], respectively; P=.11)  Greater levels of objective kidney disease knowledge also trended with more self-care behaviours, and with a stronger association observed among patients with adequate HL than those with inadequate HL |
| Balhara *et al.[24]*  2020  Cross-sectional with control  49 | Haemodialysis  Patients who had already participated, were deemed critically ill by Emergency Department clinicians, or were unable to consent, <18 | Cases -  [54, 13]  Controls - [55, 11] | 27:22 | Rapid Estimate of Adult Literacy in Medicine Short Form (REALM-SF) | To identify the specific medical factors and social determinants of health associated with missing haemodialysis and subsequent emergency department utilisation | Missed HD sessions resulting in emergency department admission | No difference in HL between cases and controls (P = 0.831) |
| Kazley *et al.[25]*  2014  Cross-sectional  127 | Advanced CKD (KTR, dialysis, pre-dialysis, pre-transplant)  Non-English speaking | 53 [17] | 61:66 | Rapid Estimate of Adult Literacy of Medicine-Transplant (REALM-T)  Newest Vital Sign (NVS) | Develop and validate a new HL tool | Decision-Making Capacity Assessment Tool (DMCAT) | Limited HL is associated with lower decision making capacity |
| Kazley *et al.[26]*  2015  Cross-sectional  92 | Advanced CKD (pre-dialysis, pre-transplant, KTR) | [54, 16] | 47:45 | Rapid Estimate of Adult Literacy of Medicine-Transplant (REALM-T)  Newest Vital Sign (NVS)  Decision-Making Capacity Assessment Tool (DMCAT) | To assess the relationship between HL and transplant outcomes | Transplant listing  Transplant received | Higher HL - more likely to be listed for transplant and to receive a transplant  A significant and positive association was found with each of the HL survey tools and the likelihood of receiving a transplant (odds ratios, 1.044, 1.672, and 1.408, respectively)  A significant and positive association was found with each of the HL survey tools and the likelihood of being listed for transplant (odds ratios, 1.020, 1.667, and 1.256, respectively)  Confounding variables: age, race, sex, marital status, insurance status |
| Gordon & Wolf*[27]*  2009  Cross-sectional  124 | KTR  Blind or severe visual impairment not correctable by eyeglasses, too ill to participate in the survey, on dialysis, <18 | [47,12] | 70:54 | Rapid Estimate of Health Literacy in Adults (REALM)  Short Test of Functional Health Literacy in Adults (S-TOFHLA) | To examine the relationship between HL levels, transplant knowledge, and graft function | Transplant function | HL levels were initially unrelated to graft function in bivariate analysis. Controlling for time after transplant greater HL was negatively correlated with serum creatinine levels with REALM-T (r =-0.20, P=.03) and S-TOFHLA (r=-0.19, P=.04).  In multivariable analysis adjusted for demographic, socioeconomic, and clinical characteristics, the relationship  between HL (S-TOFHLA) was not significant. Scores on the REALM-T significantly and independently predicted creatinine levels (β = -.03, 95% CI -.05 to -.00; P=.03) |
| Weng *et al.[28]*  2013  Cross-sectional  252 | KTR    Other transplant, <6 months post-transplant, non-English speaking, unable to give consent, <18 | 55, 45-63 | 151:101 | Short Test of Functional Health Literacy in Adults  (STOFHLA) | Prevalence and correlates of medication non-adherence | Immunosuppressive Therapy Adherence Scale (ITAS) | HL was not associated with non-adherence to medication (OR 1.1 95%CI 0.93-1.3, P=.26) |
| Ricardo *et al.[29]*    2014  Cross-sectional  2,340 | CKD 2-4  Diagnosis of polycystic kidney disease, active immunosuppression for glomerulonephritis, <21 | Limited HL [66, 9]  Adequate HL  [62, 11] | Limited HL: 221:160  Adequate HL: 1041:918 | Short Test of Functional Health Literacy in Adults  (STOFHLA) | Association of HL with kidney function and prevalence of cardiovascular risk factors | Kidney function  Self-reported CVD  BP < 130/80 mmHg  Glycated haemoglobin <7%  LDL cholesterol ≤ 100 mg/dL | In unadjusted analysis, limited HL was associated with lower kidney function, less likelihood of BP <130/80 mmHg, more self-reported CVD, lower METs, diabetes incidence  In adjusted analysis, those with limited HL were more likely to report history of CVD (OR 1.51, 95% CI, 1.13 – 2.03), and have lower kidney function (p=.03)  Adjusted for age, gender, race/ethnicity, clinical centre, education, current smoking and BMI |
| Adeseun *et al.[30]*  2012  Cross-sectional  72 | Haemodialysis or peritoneal dialysis  Previous coronary revascularisation, cardiac devices, weight >350 lbs | [51.6] | 48:23 | Short Test of Functional Health Literacy in Adults  (STOFHLA) | To examine the association between HL and risk factors for CVD including BP, lipid profile, WHR, BMI, and tobacco utilization | BP  Lipid profile  WHR  BMI  Tobacco utilization | In unadjusted analyses, there was a statistically significant  association between HL and both DBP and MAP (DBP: β −12.4, s.e. 3.4, P < 0.001; MAP: β −12.3, s.e. 3.8,  P=.002) but not SBP (β −11.9, s.e. 5.6, P=.04)  After adjusting for both demographic and socioeconomic  variables, the relationship between HL and diastolic and MAP remained statistically significant and increased in magnitude (β −13.8, s.e. 4.1, P=.001 and β −14.8, s.e. 4.6, P=.002, respectively) while the relationship with SBP reached borderline statistical significance (β −16.8, s.e. 6.7, P=.01). There was no statistically significant association between health literacy and either LDL or triglycerides.  HL was not associated with WHR, BMI, or tobacco utilization in adjusted analyses. DBP and MAP remained significant. Covariates: age, gender, race, income and educational attainment |
| Grubbs *et al.[31]*  2009  Cohort study  62 | Haemodialysis  Cognitive impairment, >75, <21, not black or white ethnicity, <9 months on dialysis | [52.4, 12.2] | 41:21 | Short Test of Functional Health Literacy in Adults (STOFHLA) | To assess the relationship between HL being listed on the waiting list for a kidney transplant | Time from dialysis start date to referral date for kidney transplant evaluation  Time from referral date to date placed on kidney transplant wait-list | No difference in mean time from dialysis start to referral date by HL (P=.60). Mean time: 3.6 (SD 6.5) months, 2.1 (SD 4.1) months for adequate HL, and 6.6 (SD 9.2) months for inadequate HL. There was a statistical difference in mean time from referral date to wait-list date by HL (P=.05)  Participants with inadequate HL had 78% lower hazard of referral for transplant evaluation than those with adequate HL after controlling for race, gender, age at start of dialysis, income, comorbid conditions, and support (adjusted hazard ratio 0.22; 95% CI: 0.08, 0.60; P=.003)  The hazard ratio of being wait-listed if referred by health literacy was not statistically different (adjusted hazard ratio 0.80; 95% CI: 0.39, 1.61; P=.50) |
| Foster *et al.[32]* 2011  Cross-sectional  238 | Haemodialysis or peritoneal dialysis  <18, unable to understand the consent process | [58, 15] | 167:144 | Short Test of Functional Health Literacy in Adults  (STOFHLA) | To examine the disaster preparedness  for dialysis-dependent patients | Disaster preparedness | Low HL was not associated with disaster preparedness (p=.52) |
| Blandon *et al.[33]*  2011  Cross-sectional  225  [abstract only] | CKD 2-4  Dialysis | 49 | 110:115 | Short Test of Functional Health Literacy in Adults  (STOFHLA) -  English or  Spanish | HL and BP control in  Hispanic Americans | BP control | Limited HL was associated with poorer BP control in women with diabetes (not in any other subgroup) |
| Dageford *et al.[34]*  2015  Cross-sectional  104 | CKD patients scheduled for an initial evaluation for kidney transplantation  Limited mental status impacting their ability to complete the phone interview, <18, non-English speaking, cognitive impairment | [52, 12] | 63:41 | Brief Health Literacy Screen (BHLS) | To evaluate characteristics of attendees and absentees of the initial transplant evaluation | Perceived knowledge about transplantation  Concerns about evaluation and transplantation | HL and other demographic and socioeconomic factors did not differ significantly between the attendees and absentees of the initial transplant evaluation |
| Warsame *et al.[35]*  2018  Cohort study  1578 | Kidney transplant candidates  Non-English speaking, <18 | [55, 13] | 964:614 | Brief Health Literacy Screen (BHLS) | To characterise HL in this population, examine whether limited HL is associated with listing for transplantation, and quantify the association between limited HL and waitlist mortality | Transplantation wait listing  Mortality [mean follow-up time 1.3 years] | Limited HL was independently associated with a decreased likelihood of listing for transplantation and increased mortality once listed  Candidates with limited HL had a 30% (aHR=0.70, 95% CI: 0.54-0.91) decreased likelihood of listing and a 2.42-fold (95% CI:1.16-5.05) increased risk of waitlist mortality  Confounding variables: age, sex, race, blood type, cause of ESKD |
| Cavanaugh *et al.[36]*  2015  Cohort  11,476  [abstract only] | Haemodialysis | Not stated | Not stated | Brief Health Literacy Screen (BHLS) | To assess International  variation in HL and association with mortality | Mortality | Low HL was significantly associated with mortality when examined as a continuous score (HR = 1.05; 95%CI = 1.03-1.08 for each point decreased) or as a category (HR = 1.65; 95%CI = 1.28-2.12 Low vs. High), and no interaction with country was observed |
| Devraj *et al.[37]*  2015  Cross-sectional  150 | CKD 1-4  Acute kidney injury, poor cognitive/visual function, Non-English speaking, <18 | Not stated  Range 21-90 | 70:80 | Newest Vital Sign (NVS) | To examine the association between HL and kidney function | Kidney function (eGFR)  Self-management knowledge | HL was associated with eGFR after controlling for all demographics except age, race and gender (which are included in eGFR equation) (P=.05)  Every unit increase in NVS score was associated with a 1.9% increase (95%CI = 0-3.86%) in eGFR (model R square = 0.23, P=.002. The relationship was non-significant after controlling for age, although it remained significant after controlling for other demographics including gender and race  No significant differences in self-management knowledge % correct by HL level were present (t = 1.27, df = 146, P=.20) |
| Devraj *et al.[38]*  2018  Cross-sectional  150 | CKD 1-4  Non-English speaking, no prior nephrology outpatient visit, acute kidney injury, cognitive impairment, poor visual acuity, <21 | Not stated  (range 21-90) | 70:80 | Newest Vital Sign (NVS) | Assess the relationship between CKD awareness, eGFR, self-management behaviours and HL | CKD awareness (CKD-A)  CKD self-management behaviour (CKD-SMB) | Multivariate analysis examining the relationship between  CKD-A and HL after controlling for demographics and  CKD-SMB knowledge. Relationship was non-significant (P > 0.05) |
| Mazarova *et al.[39]*  2017  Cross-sectional  56 | Haemodialysis  Haemodialysis <6 months, Non-English/Spanish speaking, mod/severe dementia (Mini-mental state examination (MMSE) <18), severe visual/hearing loss, medical reason not to have an fistula/graft (planned modality change, no suitable vessels, too unwell, expected death) | [63, 16] | 35:21 | Newest Vital Sign (NVS) | To describe the HL of the haemodialysis population and its association with vascular access choice | Vascular access (line vs fistula/graft)  Potassium  Phosphate  Inter dialytic weight gain | NVS score was not associated with control of K (OR 0.95 [0.30-3.06]), Phosphate [OR 1.05 (0.36-3.03)], or inter-dialytic weight gain (IDWG) (OR 1.34 [0.44-4.07])  Patients with lower HL were more likely to choose central venous catheter even if considered a good candidate for fistula/graft |
| Levine R *et al.[40]*  2018  Retrospective cohort study  142 | CKD 2-5 (including RTR and dialysis)  Non-English fluency, cognitive impairment, <12, >31 | [21, 6] | 89:53 | Newest Vital  Signs (NVS) | To examine associations between limited/inadequate HL and health services utilization among  adolescents and young adults | Health service utilisation | No association between HL and emergency department visits, total hospitalisations, preventable hospitalisations, or length of hospital stay |
| Lai *et al.[41]*  2013  Cross-sectional  63 | Haemodialysis  <6 months haemodialysis, unable to understand written and/or spoken English, severe visual and/or hearing impairment, mental and/or emotional disorders, <21 | [57, 10] | 38:25 | Functional, Communicative and Critical Health Literacy  (FCCHL) | To identify the relationships between HL and self-management  behaviours | Diabetes self-management behaviours | Total HL was found to have significant positive correlations with the total score for diabetes self-management r = 0.35; p=.005  Total HL associated with the self-care sub domains of blood glucose testing (p=0.012), foot care (p=0.010) and exercise (p=0.038)  Communicative and critical HL were associated with self-management, whereas functional HL was not |
| Indino *et al.[42]*  2019  Cross-sectional  42 | Haemodialysis  Unable to provide informed consent due to cognitive impairment, Non-English speaking, visual or hearing impairment | [54, 14] | 25:17 | Functional, Communicative and Critical Health Literacy tool (FCCHL) | To determine the association between a multidimensional concept of HL and self-reported treatment adherence | Treatment adherence | Higher overall HL was associated with increased adherence to food (OR 3.66; 95% CI 1.08-12.43; P=.038), fluid (OR 4.92; 95% CI 1.13-21.35; P=.033) and medications (OR 11.88; 95% CI 2.26-62.44; P=.003) |
| Photharos *et al.[43]*  2018  Cross-sectional  275 | CKD 2-3  eGFR <31, >90, kidney tumour or infection, able to understand and communicate in Thai, <20, >65 | Not stated    >70% were aged 51-65 years | 165:110 | Health Literacy Scale-14 (HLS-14) | To develop and test the causal relationships among family functioning, HL, CKD self-efficacy illness perceptions, social support, illness perceptions and self-management behaviours | CKD self-efficacy (CKD self-efficacy questionnaire)  Social Support (Social Support from Family and Health Provider Questionnaire)  Self-management (CKD Self-management questionnaire) | In the model explaining self-management (R^2^= 0.31)  HL: Direct effects 0.37, Total Effects 0.38, p<.050 |
| Chen *et al.[44]*    2018  Cross-sectional  410 | CKD 1-5  Paralysis, dementia, intellectual disabilities, cancer, not Chinese or Taiwanese speaking, <20 | [70, 13] | 259:151 | Short-form Mandarin Health Literacy Scale (S-MHLS) | To investigate the relationships among social support, HL, and self-management, and the factors influencing self-management | The kidney disease self-management instrument (CKD-SM) | Social support (r = .64; P <.001) and HL (r = .33; P < .001) were both positively correlated with self- management behaviours  As it had the highest explanatory power, social support was deemed the strongest predictor of self-management behaviours |
| Yu *et al*.[45]  2021  Cross-sectional  208 | CKD 1-5  No RRT | [63.2, 12.8] | 123:85 | Mandarin Multidimensional Health Literacy  Questionnaire (MMHLQ) | To examine  the relationships between different domains of self-care behaviour and HL in patients  with CKD | CKD Self-Care (CKDSC) scale | Among the five domains of self-care behaviour, HL was positively correlated with diet (β=0.15, p<0.005) and exercise (β=0.11, p=0.004) in the multivariate analysis. The patients with sufficient or excellent HL had higher diet (β=1.80, p=0.003), exercise (β=1.22, p=0.02) and home blood pressure monitoring (β=1.03, p=0.03) performance than those with inadequate or limited/problematic HL. There were no significant correlations between HL and smoking habit or medication adherence |
| Taylor *et al.[46]*  2019  Prospective cohort study  2274 | Incident dialysis  >90 days on dialysis, inability to provide consent, <18, >75 | Limited HL 58, 47-66  Adequate HL  58, 47-67 | Limited HL- 231:128  Adequate HL- 1243:672 | Single Item Literacy Screener (SILS) | To identify independent associations between HL and clinical outcomes | Time to transplant listing (deceased donor)  Time to transplantation  Survival [2 years from dialysis start] | In fully adjusted analyses (age, sex, socioeconomic status (educational level and car ownership), ethnicity, first language, primary renal diagnosis, and comorbidity, limited HL was not associated with mortality, late presentation to nephrology, dialysis modality, haemodialysis vascular access, or pre-emptive kidney transplant listing, but was associated with reduced likelihood of listing for a deceased-donor transplant (HR 0.68; 95% confidence interval [CI] 0.51- 0.90), receiving a living-donor transplant (HR 0.41; 95% CI 0.19-0.88), or receiving a transplant from any donor type (HR 0.65; 95% CI 0.44-0.96) |
| Wong *et al.[47]*  2018  Cross-sectional  137 | CKD 3-5  Non-English, Spanish or Cantonese speaking, KTR, pregnancy, dialysis, presence of co-morbid conditions that impeded meaningful communication between providers and patients, expected life expectancy <12 months, self-reported hearing impairment or severe visual impairment | [55, 12] | 66:71 | A validated 3-item screening questionnaire | To examine the relationship between HL and participation in selected self-care behaviours | Measures of selected self-care behaviours:  Medication adherence Tobacco use  Physical activity  Fast food consumption Sugary beverage intake | HL not associated with lower medication adherence (adjusted odds ratio=0.84; 95% CI 0.38-1.89) or physical activity (adjusted odds ratio=2.39; 95% CI 0.54-10.53)  Patients with lower HL had no-statistically significant higher tobacco use compared to those with adequate HL (adjusted odds ratio=2.33; 95% CI 0.90-6.06)  Individuals with low HL reported lower consumption of sugary beverages (adjusted odds ratio=0.50; 95% CI 0.20-1.23) and statistically significant lower fast food intake (adjusted odds ratio=0.38; 0.16-0.93) |
| Kita *et al.[48]*  2021  Cross-sectional  200 | CKD 3-5  Cognitive impairment or mental illness, <20 | 73 (61–80) | 128:146 | Health Literacy Survey Questionnaire (HLSEU-  Q47) | Clarify the actual condition of HL and the factors related to HL in patients with predialysis CKD | Kidney function  Albumin (g/dL)  Hemoglobin (g/dL)  Exercise habit (1 h or more per day/30 min–1 h per day/less than  30 min per day)  Daily walking time | In multivariate analysis, both social activities [Odds ratio (95% Confidence interval); 2.12 (1.16–3.89), p = 0.015] and exercise habit [Odds ratio (95% Confidence interval); 2.39 (1.16–4.90), p = 0.018] were extracted as significant factors associated with HL  No association between HL and kidney function, albumin, and haemoglobin |

*Abbreviations: BMI, body mass index; BP, blood pressure; CKD, chronic kidney disease; CVD, cardiovascular disease; DBP, diastolic blood pressure; eGFR, estimated Glomerular Filtration Rate; ESKD, end stage kidney disease; HDL, high density lipoprotein; HRQoL, health related quality of life; KTR, kidney transplant recipients; LDL, low density lipoprotein; MAP, mean arterial pressure; MET, metabolic equivalent; QoL, quality of life; RRT, renal replacement therapy; SBP, systolic blood pressure; WHR, waist to hip ratio;*

**References**

1. Dahl KG, Andersen MH, Urstad KH, Falk RS, Engebretsen E, Wahl AK. Identifying Core Variables Associated With Health Literacy in Kidney Transplant Recipients. Progress in transplantation (Aliso Viejo, Calif). 2020;30(1):38-47. h
<ttps://dx.doi.org/10.1177/1526924819893285>

2. Stomer E, Hjorthaug Urstad K, Gunnar Goransson L, Klopstad Wahl A. Health Literacy in Kidney Disease: Associations with Quality of Life and Adherence. Journal of renal care. 2020 Jun 2020;46(2):85-94. PubMed PMID: 630642213. 10.1111/jorc.12314

3. Demian MN, Shapiro JR, Thornton WL. An observational study of health literacy and medication adherence in adult kidney transplant recipients. Clinical kidney journal. 2016;9(6):858-65. h
<ttp://ovidsp.ovid.com/ovidweb.cgi?T=JS&PAGE=reference&D=prem2&NEWS=N&AN=27994867>

4. Gardiner H. The effects of health literacy on treatment adherence and health optimism in chronic kidney disease patients. 2019.

5. Murali K, Mullan J, Roodenrys S, Lonergan M. Comparison of health literacy profile of patients with end-stage kidney disease on dialysis versus non-dialysis chronic kidney disease and the influencing factors: a cross-sectional study. BMJ Open. 2020;10(10):e041404. 10.1136/bmjopen-2020-041404

6. Dawson J, Hoffman A, Josland E, Smyth A, Brennan F, Brown M. Evaluation of health literacy in end-stage kidney disease using a multi-dimensional tool. Renal Society of Australasia Journal. 2020;16(2):36-43. PubMed PMID: 145118932. Language: English. Entry Date: In Process. Revision Date: 20200815. Publication Type: Article. Journal Subset: Australia & New Zealand. 10.33235/rsaj.16.2.36-43

7. Dodson S, Osicka T, Huang L, McMahon LP, Roberts MA. Multifaceted Assessment of Health Literacy in People Receiving Dialysis: Associations With Psychological Stress and Quality of Life. Journal of health communication. 2016;21(sup2):91-8. h
<ttp://ovidsp.ovid.com/ovidweb.cgi?T=JS&PAGE=reference&D=med13&NEWS=N&AN=27683959>

8. Griva K, Yoong RKL, Nandakumar M, Rajeswari M, Khoo EYH, Lee VYW, et al. Associations between health literacy and health care utilization and mortality in patients with coexisting diabetes and end-stage renal disease: A prospective cohort study. British journal of health psychology. 2020;25(3):405-27. h
<ttps://dx.doi.org/10.1111/bjhp.12413>

9. Skoumalova I, Kolarcik P, Madarasova Geckova A, Rosenberger J, Majernikova M, Klein D, et al. Is Health Literacy of Dialyzed Patients Related to Their Adherence to Dietary and Fluid Intake Recommendations? International journal of environmental research and public health. 2019;16(21). h
<ttps://dx.doi.org/10.3390/ijerph16214295>

10. Zavacka M, Skoumalova I, Geckova AM, Rosenberger J, Zavacky P, Pobehova J, et al. Does Health Literacy of Hemodialyzed Patients Predict the Type of Their Vascular Access? A Cross-Sectional Study on Slovak Hemodialyzed Population. International journal of environmental research and public health. 2020;17(2). h
<ttps://dx.doi.org/10.3390/ijerph17020675>

11. Lim JH, Daud ZAM, Zakaria NF, Chinna K, Karupaiah T. SUN-313 EXPLORING THE RELATIONSHIPS BETWEEN HEALTH LITERACY, DIETARY ADHERENCE AND ITS MEDIATORS IN HEMODIALYSIS PATIENTS IN MALAYSIA. Kidney International Reports. 2019 Jul 2019;4(7). PubMed PMID: 2002179765. 10.1016/j.ekir.2019.05.720

12. Cavanaugh KL, Wingard RL, Hakim RM, Eden S, Shintani A, Wallston KA, et al. Low health literacy associates with increased mortality in ESRD. Journal of the American Society of Nephrology : JASN. 2010;21(11):1979-85. h
<ttps://dx.doi.org/10.1681/ASN.2009111163>

13. Tohme F, Mor MK, Pena-Polanco J, Green JA, Fine MJ, Palevsky PM, et al. Predictors and outcomes of non-adherence in patients receiving maintenance hemodialysis. International urology and nephrology. 2017;49(8):1471-9. h
<ttps://dx.doi.org/10.1007/s11255-017-1600-4>

14. Wright Nunes JA, Anderson CAM, Greene JH, Ikizler TA, Cavanaugh KL. Results of a novel screening tool measuring dietary sodium knowledge in patients with chronic kidney disease. BMC Nephrology. 2015 2015/03/31;16(1):42. 10.1186/s12882-015-0027-3

15. Jain D, Sheth H, Green JA, Bender FH, Weisbord SD. Health literacy in patients on maintenance peritoneal dialysis: prevalence and outcomes. Peritoneal dialysis international : journal of the International Society for Peritoneal Dialysis. 2015;35(1):96-8. h
<ttps://dx.doi.org/10.3747/pdi.2013.00211>

16. Patzer RE, Serper M, Reese PP, Przytula K, Koval R, Ladner DP, et al. Medication understanding, non-adherence, and clinical outcomes among adult kidney transplant recipients. Clinical transplantation. 2016;30(10):1294-305. h
<ttps://dx.doi.org/10.1111/ctr.12821>

17. Green JA, Mor MK, Shields AM, Sevick MA, Palevsky PM, Fine MJ, et al. Prevalence and demographic and clinical associations of health literacy in patients on maintenance hemodialysis. Clinical journal of the American Society of Nephrology : CJASN. 2011;6(6):1354-60. h
<ttps://dx.doi.org/10.2215/CJN.09761110>

18. Green JA, Mor MK, Shields AM, Sevick MA, Arnold RM, Palevsky PM, et al. Associations of health literacy with dialysis adherence and health resource utilization in patients receiving maintenance hemodialysis. American journal of kidney diseases : the official journal of the National Kidney Foundation. 2013;62(1):73-80. h
<ttps://dx.doi.org/10.1053/j.ajkd.2012.12.014>

19. Wright JA, Wallston KA, Elasy TA, Ikizler TA, Cavanaugh KL. Development and results of a kidney disease knowledge survey given to patients with CKD. American journal of kidney diseases : the official journal of the National Kidney Foundation. 2011;57(3):387-95. h
<ttps://dx.doi.org/10.1053/j.ajkd.2010.09.018>

20. Cavanaugh KL, Rothman RL, Wright JA, Ikizler TA. Limited health literacy associated with catheter use for chronic hemodialysis. American Journal of Kidney Diseases. 2010 Apr 2010;55(4). PubMed PMID: 70124721. 10.1053/j.ajkd.2010.02.071

21. Nelson J, Yule C, Berger A, Green J, Weisbord S. Association of health literacy with medication self-management. American Journal of Kidney Diseases. 2015 Apr 2015;65(4). PubMed PMID: 71875160. h<ttp://www.uhl-library.nhs.uk/directpages/uhlblarticles.html:> h
<ttp://www.uhl-library.nhs.uk/directpages/uhlarticles.html>

22. Singla A, Parikh R, Kreimerman J, Melamed ML, Cavanaugh KL. Associations of poor health literacy and CKD outcomes in the Bronx. American Journal of Kidney Diseases. 2016 May 2016;67(5). PubMed PMID: 72313637. h<ttp://www.uhl-library.nhs.uk/directpages/uhlblarticles.html:> h
<ttp://www.uhl-library.nhs.uk/directpages/uhlarticles.html>

23. Schrauben SJ, Cavanaugh KL, Fagerlin A, Ikizler TA, Ricardo AC, Eneanya ND, et al. The Relationship of Disease-Specific Knowledge and Health Literacy With the Uptake of Self-Care Behaviors in CKD. Kidney international reports. 2020;5(1):48-57. h
<ttps://dx.doi.org/10.1016/j.ekir.2019.10.004>

24. Balhara KS, Fisher L, El Hage N, Ramos RG, Jaar BG. Social determinants of health associated with hemodialysis non-adherence and emergency department utilization: a pilot observational study. BMC nephrology. 2020;21(1):4. h
<ttps://dx.doi.org/10.1186/s12882-019-1673-7>

25. Kazley AS, Jordan J, Simpson KN, Chavin K, Rodrigue J, Baliga P. Development and testing of a disease-specific health literacy measure in kidney transplant patients. Progress in transplantation (Aliso Viejo, Calif). 2014;24(3):263-70. h
<ttps://dx.doi.org/10.7182/pit2014958>

26. Kazley ASH, Jessica Jordan: Simpson, Kit N.: Chavin, Ken: Baliga, Prabhakar. Health literacy and kidney transplant outcomes. Progress in transplantation (Aliso Viejo, Calif). 2015;25(1):85-90. h
<ttps://dx.doi.org/10.7182/pit2015463>

27. Gordon EJ, Wolf MS. Health literacy skills of kidney transplant recipients. Progress in transplantation (Aliso Viejo, Calif). 2009;19(1):25-34. h
<ttp://ovidsp.ovid.com/ovidweb.cgi?T=JS&PAGE=reference&D=med7&NEWS=N&AN=19341060>

28. Weng FL, Chandwani S, Kurtyka KM, Zacker C, Chisholm-Burns MA, Demissie K. Prevalence and correlates of medication non-adherence among kidney transplant recipients more than 6 months post-transplant: a cross-sectional study. BMC nephrology. 2013;14:261. h
<ttps://dx.doi.org/10.1186/1471-2369-14-261>

29. Ricardo AC, Yang W, Lora CM, Gordon EJ, Diamantidis CJ, Ford V, et al. Limited health literacy is associated with low glomerular filtration in the Chronic Renal Insufficiency Cohort (CRIC) study. Clinical nephrology. 2014;81(1):30-7. h
<ttps://dx.doi.org/10.5414/CN108062>

30. Adeseun GA, Bonney CC, Rosas SE. Health literacy associated with blood pressure but not other cardiovascular disease risk factors among dialysis patients. American journal of hypertension. 2012;25(3):348-53. h
<ttps://dx.doi.org/10.1038/ajh.2011.252>

31. Grubbs V, Gregorich SE, Perez-Stable EJ, Hsu C-Y. Health literacy and access to kidney transplantation. Clinical journal of the American Society of Nephrology : CJASN. 2009;4(1):195-200. h
<ttps://dx.doi.org/10.2215/CJN.03290708>

32. Foster M, Brice JH, Shofer F, Principe S, Dewalt D, Falk R, et al. Personal disaster preparedness of dialysis patients in North Carolina. Clinical journal of the American Society of Nephrology : CJASN. 2011;6(10):2478-84. h
<ttps://dx.doi.org/10.2215/CJN.03590411>

33. Blandon J, Ling J, Alhamad T, Hernandez G. Health literacy and blood pressure control among hispanic americans with chronic kidney disease: a report from the Paso del Norte Kidney Disease Study (PNKDS). J Am Soc Nephrol. 2011;22:698A.

34. Dageforde LA, Box A, Feurer ID, Cavanaugh KL. Understanding Patient Barriers to Kidney Transplant Evaluation. Transplantation. 2015;99(7):1463-9. h
<ttps://dx.doi.org/10.1097/TP.0000000000000543>

35. Warsame F, Haugen CE, Ying H, Garonzik-Wang JM, Desai NM, Hall RK, et al. Limited health literacy and adverse outcomes among kidney transplant candidates. American journal of transplantation : official journal of the American Society of Transplantation and the American Society of Transplant Surgeons. 2019;19(2):457-65. h
<ttps://dx.doi.org/10.1111/ajt.14994>

36. Cavanaugh KL, Wang M, Wallston K, Rothman R, AlSahow A, De Sequera P, et al. International variation of characteristics of health literacy and its association with mortality in dialysis. J Am Soc Nephrol. 2015;26.

37. Devraj R, Borrego M, Vilay AM, Gordon EJ, Pailden J, Horowitz B. Relationship between Health Literacy and Kidney Function. Nephrology (Carlton, Vic). 2015;20(5):360-7. h
<ttps://dx.doi.org/10.1111/nep.12425>

38. Devraj R, Borrego ME, Vilay MA, Pailden J, Horowitz B. Awareness, self-management behaviors, health literacy and kidney function relationships in specialty practice. World journal of nephrology. 2018;7(1):41-50. h
<ttps://dx.doi.org/10.5527/wjn.v7.i1.41>

39. Mazarova A, Hiremath S, Sood MM, Clark EG, Brown PA, Bugeja AL, et al. Hemodialysis Access Choice: Impact of Health Literacy. Health literacy research and practice. 2017;1(3):e136-e44. h
<ttps://dx.doi.org/10.3928/24748307-20170711-01>

40. Levine R, Javalkar K, Nazareth M, Faldowski RA, de Ferris MD-G, Cohen S, et al. Disparities in Health Literacy and Healthcare Utilization among Adolescents and Young Adults with Chronic or End-stage Kidney Disease. Journal of pediatric nursing. 2018;38:57-61. h
<ttps://dx.doi.org/10.1016/j.pedn.2017.10.008>

41. Lai AY, Ishikawa H, Kiuchi T, Mooppil N, Griva K. Communicative and critical health literacy, and self-management behaviors in end-stage renal disease patients with diabetes on hemodialysis. Patient education and counseling. 2013;91(2):221-7. h
<ttps://dx.doi.org/10.1016/j.pec.2012.12.018>

42. Indino K, Sharp R, Esterman A. The effect of health literacy on treatment adherence in maintenance haemodialysis patients: a cross-sectional study. Renal Society of Australasia Journal. 2019;15(1):11-8. PubMed PMID: 135343045. Language: English. Entry Date: 20190319. Revision Date: 20190506. Publication Type: Article. 10.33235/rsaj.15.1.11-18

43. Photharos N, Wacharasin C, Duongpaeng S. Model of Self-management Behavior in People Experiencing Early Stage Chronic Kidney Disease. Pacific Rim International Journal of Nursing Research. 2018;22(4):360-71. PubMed PMID: 132186439. Language: English. Entry Date: 20181010. Revision Date: 20190212. Publication Type: Article. h
<ttp://search.ebscohost.com/login.aspx?direct=true&db=rzh&AN=132186439&site=ehost-live>

44. Chen Y-C, Chang L-C, Liu C-Y, Ho Y-F, Weng S-C, Tsai T-I. The Roles of Social Support and Health Literacy in Self-Management Among Patients With Chronic Kidney Disease. Journal of nursing scholarship : an official publication of Sigma Theta Tau International Honor Society of Nursing. 2018;50(3):265-75. h
<ttps://dx.doi.org/10.1111/jnu.12377>

45. Yu PS, Tsai YC, Chiu YW, Hsiao PN, Lin MY, Chen TH, et al. The Relationship between Subtypes of Health Literacy and Self-Care Behavior in Chronic Kidney Disease. J Pers Med. 2021 May 22;11(6). PubMed PMID: 34067267. PMCID: PMC8224639. Epub 2021/06/03. eng. 10.3390/jpm11060447

46. Taylor DM, Bradley JA, Bradley C, Draper H, Dudley C, Fogarty D, et al. Limited health literacy is associated with reduced access to kidney transplantation. Kidney international. 2019;95(5):1244-52. h
<ttps://dx.doi.org/10.1016/j.kint.2018.12.021>

47. Wong KK, Velasquez A, Powe NR, Tuot DS. Association between health literacy and self-care behaviors among patients with chronic kidney disease. BMC nephrology. 2018;19(1):196. <https://dx.doi.org/10.1186/s12882-018-0988-0>

48. Kita Y, Machida S, Shibagaki Y, Sakurada T. Fact-finding survey on health literacy among Japanese predialysis chronic kidney disease patients: a multi-institutional cross-sectional study. Clin Exp Nephrol. 2021 Mar;25(3):224-30. PubMed PMID: 33063230. Epub 2020/10/17. eng. 10.1007/s10157-020-01982-w
